# mRNA vaccine with unmodified uridine induces robust type I interferon-dependent anti-tumor immunity in a melanoma model

**DOI:** 10.3389/fimmu.2022.983000

**Published:** 2022-10-14

**Authors:** Chutamath Sittplangkoon, Mohamad-Gabriel Alameh, Drew Weissman, Paulo J. C. Lin, Ying K. Tam, Eakachai Prompetchara, Tanapat Palaga

**Affiliations:** ^1^ Graduate Program in Biotechnology, Faculty of Science, Chulalongkorn University, Bangkok, Thailand; ^2^ Center of Excellence in Immunology and Immune-Mediated Diseases, Chulalongkorn University, Bangkok, Thailand; ^3^ Division of Infectious Diseases, University of Pennsylvania Perelman School of Medicine, Philadelphia, PA, United States; ^4^ Acuitas Therapeutics, Vancouver, BC, Canada; ^5^ Center of Excellence in Vaccine Research and Development (Chula Vaccine Research Center-Chula VRC), Faculty of Medicine, Chulalongkorn University, Bangkok, Thailand; ^6^ Department of Laboratory Medicine, Faculty of Medicine, Chulalongkorn University, Bangkok, Thailand; ^7^ Department of Microbiology, Faculty of Science, Chulalongkorn University, Bangkok, Thailand

**Keywords:** mRNA vaccine, type I interferon, cancer immunotherapy, melanomas, unmodified nucleosides

## Abstract

An mRNA with unmodified nucleosides induces type I interferons (IFN-I) through the stimulation of innate immune sensors. Whether IFN-I induced by mRNA vaccine is crucial for anti-tumor immune response remains to be elucidated. In this study, we investigated the immunogenicity and anti-tumor responses of mRNA encoding tumor antigens with different degrees of N1-methylpseudouridine (m1Ψ) modification in B16 melanoma model. Our results demonstrated that ovalbumin (OVA) encoding mRNA formulated in a lipid nanoparticle (OVA-LNP) induced substantial IFN-I production and the maturation of dendritic cells (DCs) with negative correlation with increasing percentages of m1Ψ modification. In B16-OVA murine melanoma model, unmodified OVA-LNP significantly reduced tumor growth and prolonged survival, compared to OVA-LNP with m1Ψ modification. This robust anti-tumor effect correlated with the increase in intratumoral CD40^+^ DCs and the frequency of granzyme B^+^/IFN-γ^+^/TNF-α^+^ polyfunctional OVA peptide-specific CD8^+^ T cells. Blocking type I IFN receptor completely reversed the anti-tumor immunity of unmodified mRNA-OVA reflected in a significant decrease in OVA-specific IFN-γ secreting T cells and enrichment of PD-1^+^ tumor-infiltrating T cells. The robust anti-tumor effect of unmodified OVA-LNP was also observed in the lung metastatic tumor model. Finally, this mRNA vaccine was tested using B16 melanoma neoantigens (*Pbk*-*Actn4*) which resulted in delayed tumor growth. Taken together, our findings demonstrated that an unmodified mRNA vaccine induces IFN-I production or the downstream signaling cascades which plays a crucial role in inducing robust anti-tumor T cell response for controlling tumor growth and metastasis.

## Introduction

Cancer immunotherapy educates immune cells to recognize tumor-derived antigens, which usually have a low immunogenicity to induce a potent antigen-specific immune response to eradicate tumor cells (1). Peptide-based cancer immunotherapy has been successfully demonstrated and several trials are ongoing (2, 3). Although tumor antigen-derived peptide immunogens for cancer therapeutic vaccines are traditionally used, there are various limitations, including the manufacturing cost and the need for strong adjuvants to induce anti-tumor immunity (4). Alternative types of cancer vaccines in clinical trials such as DNA vaccines (5), autologous patient-derived immune cell vaccines (6), tumor antigen-expressing recombinant virus vaccines (7), and heterologous whole cell vaccines derived from established human tumor cell lines (8) have been reported. Prior to the widely use of mRNA vaccine for COVID-19, the mRNA-based cancer vaccines have been tested in clinical trials (9) such as personalized RNA mutanome vaccine (10) that is highly effective in inducing anti-tumor immunity. With the current use of mRNA vaccines worldwide to control the COVID-19 pandemic, the efficacy and safety of this type of vaccine platform was demonstrated (11).

The benefits of mRNA-based vaccines have been demonstrated over conventional and DNA-based vaccines. These include the safety of mRNA that will not undergo genome integration and the relatively low production cost as *in vitro* transcribed (IVT) mRNA is relatively easy to produce with a scalable manufacturing process (11). mRNAs can be prepared for any protein antigen by using host cell’s translational machinery avoiding MHC restriction in contrast to peptide vaccines (12, 13). The short half-life of mRNA and transient antigen expression enables repeated administration alleviating potential issues such as the low risk of immune suppression due to chronic persistence of antigens (14). Furthermore, mRNA can efficiently transfect nondividing cells (15) and may be engineered and manufactured to provide self-adjuvanticity (16).

Over the past decades, obstacles in applying mRNA as vaccines including the inherent instability and low level of protein expression as a result of high innate immunogenicity and inefficient delivery have been resolved (17). Purified unmodified IVT mRNA induces high level of type I interferon (IFN-I) through activation of toll-like receptors (TLRs). Activation of TLRs results in upregulation of proinflammatory cytokines such as IFN-I, IL-6, IL-12, TNF-α and chemokines (18). This can be circumvented by the incorporation of naturally occurring nucleosides and by applying stringent purification to remove double-stranded RNA contaminants (19–21). Karikó et al. reported that mRNA with nucleoside modifications were insensitive to ribonuclease L (RNase L) degradation, did not activate TLRs and protein kinase R, which subsequently resulted in improved translational efficiency and stability, compared to unmodified mRNA (20). On the other hand, Thess et al. reported that codon-optimized unmodified mRNA showed higher translational efficiency with low immunogenicity compared to the modified mRNA (22). This study, however, engineered the regulatory regions of the construct so that the internal ribosome entry site was modified, reducing ribosome binding leading to less efficient translation.

Due to the heterogeneity of tumor antigens as a result of high mutation rates, neo-antigens with tumor-specific mutations are ideal targets for cancer immunotherapy as they can potentially be recognized by the mature T-cell repertoire as non-self antigens (23). Recently, nonsynonymous somatic point mutations in B16F10 murine melanoma cells have been reported. Using this dataset, tumor-specific mutations were identified by algorithms and selected vaccine targets were based on their expression levels and major histocompatibility complex (MHC) binding capacity (24, 25). Surprisingly, when immunized with IVT mRNA, most of the mutation-specific immune response biased toward mutation-specific CD4^+^ T cells. Repeated vaccination of mRNA encoding a lysine to asparagine (K739N) mutation in the *Kif18b* slowed tumor growth, prolonged survival, and inhibited lung metastasis in B16F10 tumor model (25). However, the impact of different degrees of nucleoside modifications on mRNA-LNP cancer vaccine-induced anti-tumor immunity has not been investigated.

In this study, we aimed to compare the anti-tumor efficacy of mRNA vaccines with various degrees of nucleoside modification in a mouse melanoma model of localized and metastatic tumor. Furthermore, the roles of IFN-I from innate immune induction by mRNA vaccine on T cell responses and therapeutic efficacy to control tumor growth were investigated. Finally, we investigated the therapeutic efficacy mRNA vaccine encoding neoantigens of PDZ-binding kinase (PBK) and actinin alpha 4 (ACTN4), which were identified from the B16F10 murine melanoma mutanomes (26).

## Materials and methods

### Animals

Wild type C57BL/6 mice were purchased from Charles River Laboratories (Wilmington, MA, USA) or Nomura Siam International (Bangkok, Thailand). Age-matched (6–12 weeks) female mice were used in all experiments. Mice were maintained in a specific pathogen-free facility, and all protocols involving laboratory animals were approved by the institutional animal care and use committee (IACUC) at the University of Pennsylvania and Chulalongkorn University (Protocol Review No. 803941; 1723013; 1873005; 003/2565). The results are reported under the Animal Research: Reporting of *In Vivo* Experiments (ARRIVE) Guidelines.

### RNA constructs, *in vitro* transcription, and lipid nanoparticle formulation

Plasmid templates for *in vitro* transcription of antigen-encoding RNAs were based on the previously published pUC-ccTEV-A101 vector (27). pUC-ccTEV-ovalbumin-A101 (OVA), pUC-ccTEV-neoantigens-A101 (Neo), pUC-ccTEV-luciferase-A101 (Luc2), pUC-ccTEV-mCherry-A101 (mCherry), and pUC-ccTEV-PR8HA-A101 (PR8HA) vectors were synthesized by GenScript (Piscataway, NJ, USA). The Neo construct contained the sequence encoding two point-mutated 27-meric peptides (Pbk and Actn4) linked by a sequence encoding a 10 amino-acid long glycine-serine linker. The mRNAs were produced from plasmids encoding codon-optimized antigens. Plasmids were linearized with restriction enzymes, mRNA was produced using the MEGAscript T7 Transcription Kit (Ambion, USA), and purified using cellulose-loaded column (28). Percent of 1-methylpseudouridine-5′-triphosphate (m1ψ) (TriLink, USA) and UTP nucleotides were varied by mole (0, 5, 10, 20, 30, 40, 50, 60, 70, 80, 90 and 100% m1ψ). RNAs were capped using CleanCap AG (3’OMe) (TriLink). All RNAs were analyzed for the integrity by native agarose gel electrophoresis and for double-stranded RNA using dot blot. mRNAs were stored frozen at −80°C until use.

Purified mRNAs were formulated into lipid nanoparticles using a self-assembly process wherein an ethanolic lipid mixture of an ionizable cationic lipid, phosphatidylcholine, cholesterol, and polyethylene glycol-lipid was rapidly combined with an aqueous solution containing mRNA at acidic pH as previously described (29). The ionizable cationic lipid (pKa in the range of 6.0-6.5, proprietary to Acuitas Therapeutics) and LNP composition are described in the patent application WO 2017/004143. The average hydrodynamic diameter was ~80 nm with a polydispersity index of 0.02-0.06 as measured by dynamic light scattering using a Zetasizer Nano ZS (Malvern Instruments Ltd, Malvern, UK) and an encapsulation efficiency of ~95% as determined using a Ribogreen assay.

### Cell transfection

Bone marrow-derived macrophages (BMDMs) were harvested after 7-days of differentiation with cold phosphate-buffered saline (PBS) and seeded 5×10^4^ cells/well in 200 µl of DMEM complete media in a 96-well plate. Bone marrow-derived dendritic cells (BMDCs) were used for transfection after 12 days of differentiation. BMDCs and BMDMs were incubated with mRNA encoding mCherry complexed with TransIT transfection reagent (0.1 µg mRNA in 17 μl TransIT transfection reagent) (Mirus Bio, USA). This complex was added to cells in 183 μl media. Reporter proteins in mRNA-transfected cultured cells were detected and quantified at 48 hr after transfection. mCherry positive cells were quantified by LSR II flow cytometer (BD Biosciences, USA).

### Generation of BMDMs

Bone marrow cells (BMs) were flushed from humerus, femur and tibia of 6-8 weeks old C57BL/6 mice. To obtain BMDMs, BMs were cultured in tissue culture non-treated petri dish (Hycon, Thailand) with 8 ml of DMEM complete media (Cytiva, UK) supplemented with 10% (v/v) Fetal Bovine Serum (FBS) (Gibco, USA), 10 mM HEPES (Cytiva), 1 mM sodium pyruvate (Cytiva) and 1x Penicillin/Streptomycin G (Cytiva), supplemented with 5% (v/v) horse serum (Cytiva), and 20% (v/v) L929-conditioned media. On day 4, three ml of fresh media supplemented with 20% L929-conditioned media and 5% horse serum was added to the culture. Cells were harvested on day 7 using ice-cold PBS. Macrophage phenotype was confirmed by flow cytometry by staining using mouse anti-F4/80 and CD11b antibodies (BioLegend, USA). The derived BMDMs were seeded at 5×10^4^ cells/well in 200 µl of DMEM complete media without horse serum and L929-conditioned media in 96-well plate before transfection.

### Generation of BMDCs

BMs were cultured in 96-well tissue culture treated plate (Nunc, USA) at 1×10^4^ cells/well in 100 µl of BMDC media containing RPMI-1640 (Cytiva) supplemented with 10% (v/v) FBS, 10 mM HEPES, 1 mM sodium pyruvate, 1x Penicillin/Streptomycin G, 1x GlutaMAX™ (Gibco), 1x MEM Non-Essential Amino Acid (Gibco), 55 µM β-mercaptoethanol (Gibco). On day 0, recombinant mouse GM-CSF (20 ng/ml) (Peprotech, USA) was added to the media. On day 3, 6, and 8, fresh BMDC media containing recombinant mouse GM-CSF (20 ng/ml) was added. On day 10, the culture supernatant was discarded and the same volume of fresh BMDC media containing recombinant mouse GM-CSF (20 ng/ml) and recombinant mouse IL-4 (10 ng/ml) (Peprotech) were added. On day 11, ¾ the volume of culture supernatant was discarded and the same volume of fresh BMDC media containing recombinant mouse GM-CSF (20 ng/ml) and recombinant mouse IL-4 (5 ng/ml) was added. The derived BMDCs were cultured in BMDC media without cytokines and used for transfection on day 12. Dendritic cell phenotype was confirmed by flow cytometry by staining with mouse anti-CD11c, MHC class II, CD40, CD86 antibodies (BioLegend).

### Tumor cell lines

B16F10-Luc2 melanoma cells were obtained from the American Type Culture Collection (ATCC CRL-6475-LUC2™). Cells were maintained in DMEM media supplemented with 10% FBS and 10 µg/ml Blasticidin (*In vivo*Gen, USA). The OVA-secreting B16F0-OVA cell line was kindly provided by Dr. Edith Lord (Univiversity of Rochester, Rochester, NY, USA). Cells were maintained in RPMI-1640 supplemented with 10% FBS, 10 mM HEPES, 1 mM sodium pyruvate, 1x GlutaMAX™, 1x MEM Non-Essential Amino Acid, 55 µM β-mercaptoethanol, 1x Penicillin/Streptomycin G and G418 (400 μg/ml) (*In vivo*Gen).

### Melanoma tumor model

For immunogenicity studies, age-matched female (8-12 weeks old) C57BL/6 mice were immunized with mRNA encoding ovalbumin or neoantigens on day 0 and boosted on day 4 with the same dose and formulation. Vaccination was performed by intramuscular (i.m.) injection of 10 µg mRNA-LNP. Mice were sacrificed 7 days after the booster vaccine for spleen collection.

For therapeutic study, anesthetized mice were injected subcutaneously (s.c.) into the flank with 2 × 10^5^ B16F0-OVA or B16F10-Luc2 tumor cells in 200 μl of sterile Hanks’ Balanced Salt Solution (Gibco). For B16F0-OVA model, two doses of 10 µg of mRNA encoding ovalbumin or irrelevant antigen were administered i.m. on day 4 and 8 after tumor inoculation. For the B16F10-Luc2 model, mice were immunized i.m. with two doses of 10 µg of mRNA encoding neoantigen or irrelevant antigen on day 4 and 8 after tumor inoculation. Tumor growth was monitored 2–3 times a week, and the survival was recorded for at least 31 days. Tumor volumes were monitored by using a vernier caliper and calculated using the equation: V = (4×3.14×A × B^2^)/3, where V = volume (mm^3^), A = the largest diameter (mm), and B = the smallest diameter (mm) (30). Mice were sacrificed when tumor size reached 20 mm in diameter or 400 mm^2^ (31).

For the cancer lung metastasis model, 2 × 10^6^ B16F0-OVA tumor cells in 200 μl of sterile Hanks’ Balanced Salt Solution were injected intravenously (i.v.) through lateral tail veins. On day 4 and 8 after tumor inoculation, mice were immunized *i.m.* with 10 µg of mRNA encoding ovalbumin or irrelevant antigen. On day 18, mice were euthanized with isoflurane. Lungs were fixed and bleached in Fekete’s solution to count the tumor nodules on the lung surface.

### ELISA

Culture supernatants from treated BMDMs and BMDCs were harvested at 48 hr after transfection as described above. Serum collected from immunized mice treated as indicated was prepared. Mouse IFN-α (Invitrogen, USA) and IFN-β ELISA (BioLegend) was carried out according to the manufacturer’s instructions.

### Synthetic peptides

For the *in vitro* re-stimulation of splenocytes, a pool of six synthetic peptides (Jerini Peptide Technologies, Germany) of 11-27 amino acids (a.a.) in length, with eight overlapping residues were used. The purity of the peptides was > 95% HPLC purified. Peptides corresponded to the mutated sequences of *Pbk* (PAAVILRDALH, VILRDALHMAR and DSGSPFPAA VILRDALHMARGLKYLHQ) and *Actn4* (FQAFIDVMSRE, FIDVMSRETTD and NHSGLVTFQAFIDVMSRETTDTDTADQ) were used as neoantigens. The peptides were used at a final concentration of 2.5 µg/ml. A synthetic peptide 8 a.a. in length from the sequence of ovalbumin (H2-K^b^-restricted OVA_257-264_ SIINFEKL) was used at a concentration of 2.5 µg/ml (*In vivo*gen, USA).

### Serum and tissue preparation

Serum prepared from peripheral blood was collected with capillary tube and were stored at -80°C until use. Spleens and draining lymph nodes were collected, and single-cell suspensions were prepared in RPMI-1640 containing 10% (v/v) FBS after filtering through 70-μm cell strainer (BD Falcon, USA). Erythrocytes were removed by ACK lysing buffer (Quality Biological, USA). Murine B16 tumors were harvested and minced into small pieces. Cells were washed once with FBS (10% (v/v)) in RPMI-1640. After centrifugation at 2,000 rpm for 10 minutes at 4°C, collagenase IV (1 mg/ml) and DNase I (100 mg/ml) (both were from Sigma-Aldrich, USA) in RPMI-1640 containing 10% (v/v) FBS, 1x Penicillin/Streptomycin G, 55 µM β-mercaptoethanol were added and incubated at 37°C for 20 minutes in 250 rpm shaking incubator to disperse aggregates. Cell suspensions were passed through 40-µm cell strainer and washed once with 2% (v/v) FBS in RPMI-1640 and centrifuged at 2,000 rpm for 10 minutes at 4°C. Erythrocytes in tumor suspension were removed by ACK lysing buffer. Cell numbers were counted with Vi-Cell XR cell counter (Beckman Coulter, USA).

### *In vitro* re-stimulation and cell surface staining and intracellular cytokine staining (ICS) of splenocytes

After *in vitro* re-stimulation of 2 × 10^6^ splenocytes with OVA_257-264_ SIINFEKL (2.5 µg/ml) or a pool of six synthetic peptides of Pbk and Actn4 neoantigens (2.5 µg/ml each) in the presence of purified anti-mouse CD28 antibody (1 μg/ml) (BioLegend) for 6 hr with brefeldin A (20 μg/ml), and GolgiStop™ (40 μg/ml) (BD Pharmingen, USA) for the last 5 hr, cells were washed in PBS and stained with the LIVE/DEAD Fixable Aqua Dead Cell Stain Kit (Life Technologies, USA) for 10 minutes at room temperature. Cells were washed once in FACS buffer containing 2% (v/v) FBS in PBS and blocked with purified anti-mouse CD16/CD32 (0.5 µg) (BD Biosciences, USA) for 20 minutes at 4°C. Monoclonal antibodies used for surface staining and ICS are shown in **Supplementary Table 1**. ICS was performed using Cytofix/Cytoperm kit (BD Biosciences). Data were acquired on an LSR Fortessa flow cytometer (BD Biosciences, USA) and analyzed with FlowJo 10.6.0 software (Tree Star, USA).

### ICS of tumor infiltrating immune cells

After single cell suspensions were prepared as stated above, cells were counted, and 2 × 10^6^ cells were stained with the LIVE/DEAD Fixable Aqua Dead Cell Stain Kit or PI (BioLegend, USA) for 10 minutes at room temperature. Cells were washed once in FACS buffer containing 2% (v/v) FBS in PBS and blocked with purified anti-mouse CD16/CD32 (0.5 µg) for 20 minutes at 4°C. Monoclonal antibodies used for surface staining and ICS are shown in **Supplementary Table 1**. ICS was performed using Cytofix/Cytoperm kit.

### Blocking IFN-I by IFNAR1-specific monoclonal antibody

B16F0-OVA cell lines were injected into mice to allow for tumor formation as described above. At day 4 and day 8, mice were intraperitoneally injected with 400 µg/dose of IFNAR1-specific MAR1-5A3 mAb (BioLegend) (32) or MOPC-21 isotype control mAb (BioLegend, USA) as described previously (33) 1 hr before mRNA-LNP vaccination (30). Tumor growth and immune infiltrated cells were analyzed at day 42.

### ELISpot

Splenocytes from immunized mice (2×10^6^ cells) were cultured for 48 hr in IFN-γ (BD Biosciences) pre-coated 96-well plates in the presence of OVA (20 μg/ml) (*In vivo*Gen) or concanavalin A (10 μg/ml) (Sigma-Aldrich). ELISpots were performed according to the manufacturer’s instructions by ELISpot plate reader (ImmunoSpot, USA).

### Statistical analysis

Statistical analyses were performed with Prism 8.0 (GraphPad Software). Data were compared with an unpaired two-tailed Student’s t test, one-way or two-way ANOVA with Bonferroni multiple comparisons test. Statistical significance was defined by a value of *P* < 0.05. The log-rank test followed by the Mantel-Cox posttest was used for the survival analysis.

## Results

### Translation efficiency is inversely related to IFN-I secretion, APC maturation and levels of nucleoside modification

**Figure 1:**
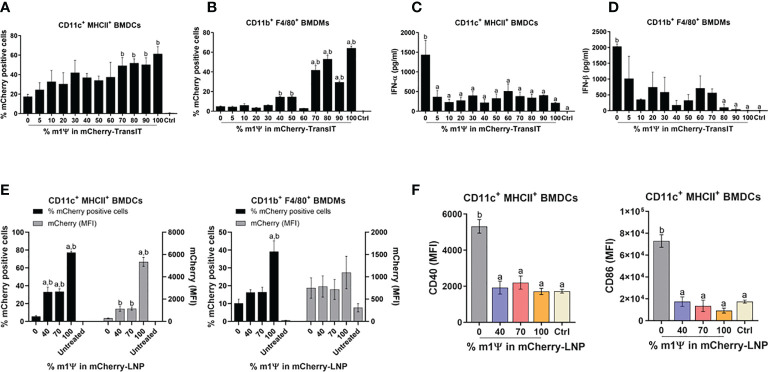
TransIT and LNP efficiently delivered mRNA to murine BMDCs and BMDMs *in vitro*. **(A–D)** mCherry-encoding mRNA modified with different % of m1Ψ (0.1 µg) were transfected into BMDCs and BMDMs using TransIT reagent. The frequencies of mCherry positive **(A)** BMDCs and **(B)** BMDMs were determined by flow cytometry at 48 hr after transfection. **(C)** The levels of IFN-α and **(D)** IFN-β released upon mRNA transfection from BMDCs and BMDMs, respectively were examined by ELISA. **(E)** mCherry-encoding mRNA modified with different % of m1Ψ (0.1 µg) were delivered into BMDCs and BMDMs using LNP. The frequencies of mCherry positive BMDCs (left) and BMDMs (right) and MFI of mCherry delivered by LNP were determined by flow cytometry. **(F)** MFI of CD40 (left) and CD86 (right) on BMDCs upon mRNA-LNP transfection was shown. The control were untranfected cells. The results are presented as the mean ± SEM of at least duplicate samples and experiments were performed at least two times. Statistical significance by one-way ANOVA with Bonferroni multiple comparisons test were indicated when *p* < 0.05 compared to the unmodified target antigen: a, or control (PBS): b.

The effect of nucleoside modified mRNA on protein translation, IFN-I production and APC maturation, were initially evaluated *in vitro* using the commercial reagent TransIT to deliver mCherry encoding mRNA with different levels of m1ψ modification (0, 5, 10, 20, 30, 40, 50, 60, 70, 80, 90, and 100%) into BMDCs and BMDMs. *In vitro* transfection of 0.1 µg mRNA with levels of m1ψ substitution in the range of 70-100% showed significantly higher percentages of mCherry^+^ cells, compared to the untreated control in both BMDCs and BMDMs at 48 hr post-transfection (**Figures 1A, B**; **Figures S1A, B**). Only mRNA with 0% of m1ψ subsitution (referred to as unmodified mRNA) showed a strong induction of IFN-I production in both cells (**Figures 1C, D**). Based on this initial result, mRNA with 0, 40, 70, and 100% of m1ψ substituting conditions were selected for LNP encapsulation in subsequent experiments.

Similar to the results obtained by TransIT reagent, LNP formulated modified mRNA with 100% of m1ψ substitution resulted in a significantly higher percentages and median fluorescence intensity (MFI) of mCherry^+^ cells than other conditions in both BMDCs and BMDMs with 77% and 39% of mCherry^+^ cells, respectively (**Figure 1E**; **Figures S1A, B**). Although modified mRNA with 100% of m1ψ substitution showed efficient protein translation, this treatment did not significantly induce maturation of BMDCs. In contrast, cells transfected with unmodified mRNA significantly upregulated CD40 and CD86 expression, suggesting DC maturation (**Figure 1F**; **Figure S1A**).

**Figure 2:**
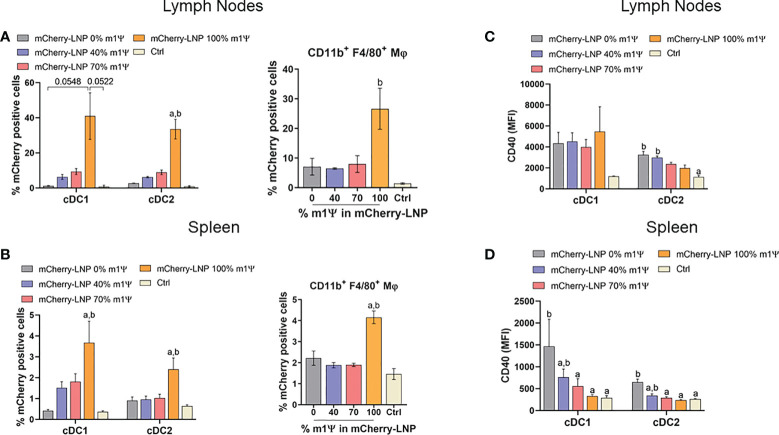
mRNA-LNP uptake by APCs in lymph nodes (LNs) and spleens *in vivo*. Mice were intramuscular injected with 10 µg of mCherry mRNA-LNP with 0, 40, 70, or 100% of m1Ψ modification and the control group received PBS. At 48 hr of mRNA administration, quantification of mCherry-positive cells in **(A)** LNs and **(B)** spleen of conventional type 1 (cDC1) and conventional type 2 (cDC2) dendritic cells (left), and MΦ (right) were determined. MFI of costimulatory molecule CD40 on cDC1 and cDC2 from **(C)** LNs and **(D)** spleen were shown. The results are presented as the mean ± SEM of biologically independent mice (*n* = 6) per group. Statistical significance by one-way ANOVA with Bonferroni multiple comparisons test when *p* < 0.05 compared to the unmodified target antigen: a, or control (PBS): **(D)** cDC1 subset was defined as Dump^-^ (B220^-^ NK1.1^-^ CD3^-^ TER-119^-^ CD19^-^) CD11c^+^ MHCII^+^ XCR1^+^. cDC2 subset was defined as Dump^-^ (B220^-^ NK1.1^-^ CD3^-^ TER-119^-^ CD19^-^) CD11c^+^ MHCII^+^ CD172a^+^.

For an *in vivo* delivery of mRNA, DCs and macrophages in draining lymph nodes (LN) (popliteal and inguinal LNs) and spleens were examined. Intramuscular administration (i.m.) of 10 µg mRNA-LNP with 100% of m1ψ substitution was efficiently taken up and translated into proteins by cDC1, cDC2 and macrophages in both LNs and spleens with the highest percentages of mCherry^+^ cells observed after 48 hr (**Figures 2A, B**; **Figure S1C**). CD40 upregulation in LN cDC1 was equally observed in all types of mRNA, regardless of m1ψ modification that is higher than the untreated control (**Figure 2C**; **Figure S1C**). However, exposure to mRNA with 0 and 40% of m1ψ substitutions significantly enhanced CD40 expression in LN cDC2 and splenic cDC1 and cDC2, respectively (**Figures 2C, D**; **Figure S1C**). Taken together, these results indicated that modified mRNA with 100% m1ψ substitutions significantly improves translation efficiency and decreases innate immunogenicity. Although unmodified mRNA compromises the translation efficiency, it induces high levels of type I IFN and robust expression of costimulatory molecules in major APCs.

### Immunization with OVA mRNA-LNP induces robust immune responses and activates OVA-specific cytotoxic effector T cells

**Figure 3:**
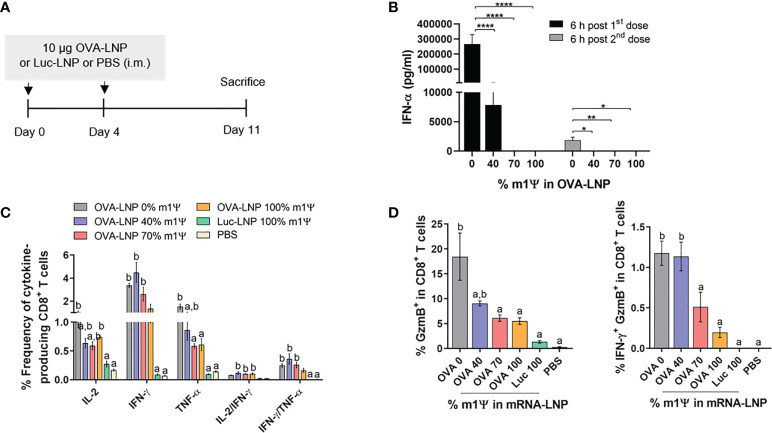
Immunization of mRNA-LNP elicits robust antigen-specific CD8^+^ T cell responses. **(A)** Schematic representation of the immunization regimen (see methods for details). **(B)** IFN-α concentration in the serum 6 hr after the first (day0) and the second (day4) immunizations of OVA mRNA-LNP were detected by ELISA. **(C, D)** The frequencies of IL-2, IFNγ, TNFα and Granzyme B (GzmB) and IFN-γ/GzmB-producing CD8^+^ T cells after 6 hr stimulation with OVA_257-264_ (SIINFEKL) were measured by flow cytometry. The results are presented as the mean ± SEM, *n* = 4-5 biologically independent mice per group. Statistical significance: Statistical significance by one-way ANOVA with Bonferroni multiple comparisons test were indicated as (*)*p* < 0.05, (**)p < 0.01 and (****)*p* < 0.0001 or *p* < 0.05 compared to the unmodified target antigen: a, or control (PBS): b.

To evaluate whether mRNA prepared with varying degrees of m1Ψ modification differentially induces antigen-specific CD8^+^ T cell responses, mice were i.m. immunized with two doses of ovalbumin encoding mRNA (OVA-LNP) (10 µg/dose) or PBS with 4 days interval between the two doses (**Figure 3A**). Luciferase encoding mRNA (Luc-LNP) was used as unrelated antigen control. Immunization with OVA-LNP with unmodified mRNA or with 40% m1Ψ modification significantly increased serum IFN-α concentration at 6 hr post first and second immunization, compared to the mRNA with m1Ψ modification of 70 and 100% (**Figure 3B**). After the second dose, the level of IFN-α induced by unmodified mRNA was much lower compared to that from the first dose but remained at detectable level.

Seven days after the second mRNA immunization, splenocytes were restimulated with CTL epitope SIINFEKL OVA peptide *in vitro*. The frequency of IL-2- and IFN-γ producing CD8^+^ T cells increased in all groups of mice receiving OVA-LNP, regardless of the level of m1Ψ modification. On the other hand TNFα-producing CD8^+^ T cells were higher in mice receiving OVA-LNP with m1Ψ modification of 0 and 40% than 70% or 100% modification (**Figure 3C**; **Figure S1D**). Significantly increased percentages of IL-2/IFN-γ-double producers were detected in the groups receiving OVA-LNP with m1Ψ substitution of 40, 70, and 100% (**Figure 3C**; **Figure S1D**). In addition, a significantly higher percentages of granzyme B and IFN-γ/granzyme B-producing CD8^+^ T cells were observed in the group with OVA-LNP with m1Ψ modification of 0 and 40%, compared to those with 70 or 100% m1Ψ substitution (**Figure 3D**; **Figure S1D**). Taken together, these results demonstrated that administration of OVA-LNP robustly activates OVA-specific CD8^+^ T cell responses, particularly unmodified mRNA effectively stimulates CTL responses.

### Unmodified mRNA induces antitumor immunity and alters tumor-infiltrating immune cell profiles

**Figure 4:**
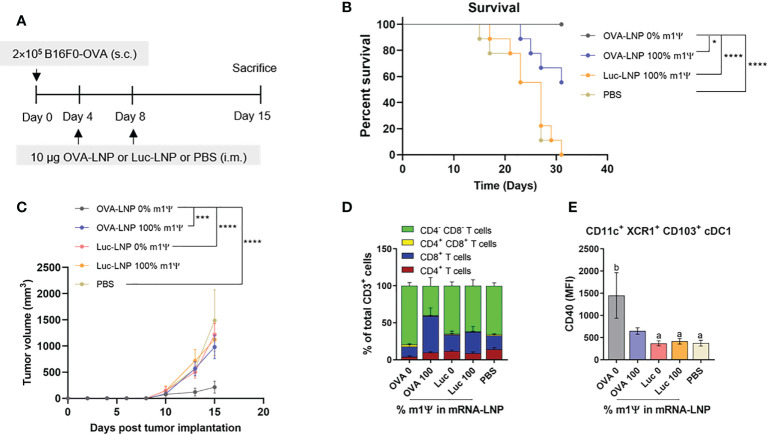
Intramuscular immunization of unmodified OVA-LNP inhibits tumor growth and prolongs survival. **(A)** Schematic representation of the immunization and tumor implantatioin schedule. **(B)** The percentages of survival mice was followed until day 31 after tumor implantation. Mice that reached the maximal allowed tumor size of 20 mm, or 400 mm^2^ were euthanized and recorded as having tumor areas of 400 mm^2^ (*n* = 9). **(C)** Tumor volume was shown during the 15 days after tumor implantation (*n* = 10). **(D)** Compositions of tumor-infiltrating T cells and **(E)** MFI of costimulatory molecule CD40 on cDC1 are shown (*n* = 6). The results are presented as the mean ± SEM. Statistical significance: (*)*p* < 0.05, (***)*p* < 0.001 and (****)*p* < 0.0001 by two-way ANOVA with Bonferroni multiple comparisons test. Survival curves were compared using log-rank (Mantel–Cox) test.

To determine whether the immune responses induced by OVA-LNP is sufficient to control tumor growth, murine B16F0-OVA melanoma expressing OVA was used as a model. Mice were transplanted s.c. with B16F0-OVA at day 0 and i.m. immunized with two doses of OVA-LNP (10 μg/dose) with unmodified and m1Ψ modification of 100% mRNA or control Luc-LNP with m1Ψ modification of 100% or PBS on day 4 and 8 (**Figure 4A**). The vaccination schedule in our studies was based on previous reports on cancer vaccines by the Fotin-Mleczek et al. sponsored by CureVac (34) and Kranz et al. (30). The schedule was designed to prevent the typical post-expansion T-cell retraction phase and maintain high frequencies of antigen-specific T cells. All animals reached termination criteria without significant weight loss (**Figure S2**). Mice immunized with unmodified OVA-LNP survived until the end of the experimental period of 31 days while all mice in the PBS or Luc-LNP control group were dead (**Figure 4B**). For OVA-LNP with m1Ψ modification of 100%, half of the mice survived. The survival rates reflected the delay and significant decrease in tumor growth in unmodified OVA-LNP groups compared with the other groups (**Figure 4C**).

To monitor the impact of mRNA vaccine on tumor infiltrated immune cells, seven days after a boost, mice were sacrificed and the tumor infiltrated immune cells (T cells and DCs) were characterized. The majority of tumor-infiltrating CD3^+^ T cell population in the unmodified OVA-LNP group were CD4^-^CD8^-^ T cells, while the group receiving OVA-LNP with m1Ψ modification of 100% had CD8^+^ T cells as the major population (**Figure 4D**; **Figure S3**). We next characterized the intratumoral migratory cDC1 (CD11c^+^ XCR1^+^ CD103^+^). In a group receiving unmodified OVA-LNP, a significant increase in CD40 level among cDC1 subset was observed (**Figure 4E**; **Figure S3**). This result strongly supports that unmodified mRNA induces more efficient DC activation that may augment the anti-tumor immunity and skews toward Th1 in the tumor microenvironment.

**Figure 5:**
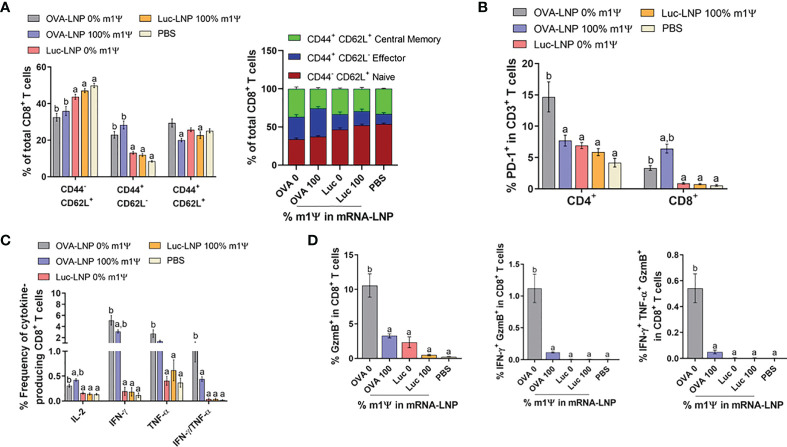
Immunization of unmodified OVA-LNP enhances the activation of antigen-specific CD8+ T cells. Mice were treated as described in **Figure 4A** and the splenocytes were re-stimulated for 6 h with OVA_257-264_ (SIINFEKL) peptide. **(A)** The frequencies of naïve cells (CD44^-^CD62L^+^), effector cells (CD44^+^CD62L^-^), and memory cells (CD44^+^CD62L^+^) in CD3^+^ CD8^+^ T cell subsets **(B)** the frequencies of PD-1+ CD4^+^ and CD8^+^ T cells and **(C)** the frequencies of cytokines-producing CD8^+^ T cells are shown. **(D)** The OVA_257-264_-specifc responses were determined and the percentages of CD8^+^ T cells producing GzmB, IFN-γ/GzmB, or IFN-γ/TNF-α/GzmB are shown. The results are presented as the mean ± SEM of biologically independent mice (n=7) per group. Statistical significance by one-way ANOVA with Bonferroni multiple comparisons test when *p* < 0.05 compared to the unmodified target antigen: a, control (PBS): b.

We next investigated the impact of OVA-LNP vaccination on phenotypes of immune cell population in the spleens on day 15 after tumor implantation and mRNA vaccine administration. Consistent with the strong anti-tumor phenotype, mice immunized with unmodified OVA-LNP or with m1Ψ modification of 100% showed significant expansion of effector CD8^+^ T cells (CD8^+^ CD44^+^ CD62L^-^). Within the memory CD8^+^ T cell population (CD8^+^ CD44^+^ CD62L^+^), mice receiving unmodified OVA-LNP showed relatively higher frequency of memory CD8^+^ T cell population than mice receiving OVA-LNP with m1Ψ modification of 100% (**Figure 5A**; **Figure S4**). This relative increase in memory CD8 T cell expansion observed in unmodified OVA-LNP group may be due to the smaller percentage of effector subset. In addition, PD-1 exhaustion marker on T cells of immunized mice was investigated. There was a significant increase in PD-1^+^ CD8^+^ T cells in the group with OVA-LNP with m1Ψ modification of 100% whereas the PD-1^+^ CD4^+^ T cells increased in the unmodified OVA-LNP group (**Figure 5B**; **Figure S4**).

We also evaluated the induction of SIINFEKL-specific CD8^+^ T cell responses. As shown in **Figure 5C**; **Figure S4**, both modified and unmodified OVA-LNP significantly induced higher frequencies of cytokine producing cells in SIINFEKL-specific CD8^+^ T cells. More importantly, compared with the modified OVA-LNP group, unmodified OVA-LNP induced higher percentages of grazyme B^+^ or IFN-γ/TNFα-double producers and granzyme B/IFN-γ-double producers in CD8^+^ T cells (**Figure 5D**; **Figure S4**). This coordinated anti-tumor immunity induced by unmodified OVA-LNP reflects the delayed tumor growth and higher survival rate in tumor transplanted animals.

### Type I interferon (IFN-I) is crucial for anti-tumor effect induced by unmodified mRNA vaccine

**Figure 6:**
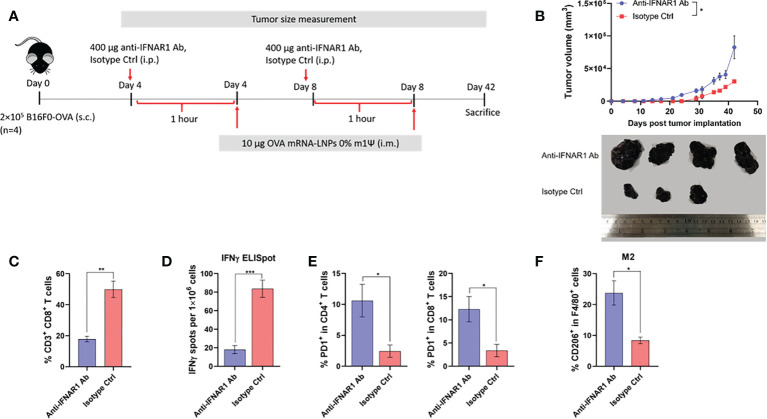
Type I IFN signaling promotes antitumor effect and modulated immune cell profile. **(A)** Scheme of immunization regimen and IFNAR1 antibody or isotype control treatment. See details in methods. **(B)** The tumor volume (top panel) and the representative tumor mass (lower panel) harvested 42 days after the tumor implantation from mice treated with anti-IFNAR1 antibody or isotype control followed by immunization with unmodified OVA-LNP. **(C)** The frequency of splenic CD8^+^ T cells was examined by flow cytometry. **(D)** ELISpot of IFN-γ producing cells among splenocytes after 48 hr of *ex vivo* re-stimulation with OVA on day 42 after tumor implantation and mRNA vaccine treatments is shown. **(E)** The frequency of PD-1^+^ cells among tumor infiltrated CD4^+^ (left) and CD8^+^ (right) T cells were examined by flow cytometry. **(F)** The frequency of tumor infiltrated CD206^+^ macrophages was determined by flow cytometry. The results are presented as the mean ± SEM, *n* = 4 biologically independent mice per group. Statistical significance: (*)*p* < 0.05, (**)*p* < 0.01, (***)*p* < 0.001 by unpaired two-tailed Student’s t test.

In order to gain an insight how unmodified mRNA induces robust anti-tumor immunity, we evaluated the role of IFN-I in the therapeutic efficacy of mRNA vaccine. Mice were implanted with B16F0-OVA on day 0, followed by intraperitoneally administration of anti-IFNAR1 antibody or isotype control (400 µg per mouse) on day 4 and day 8. One hour after the administration of anti-IFNAR1 antibody or isotype control, mice were i.m. immunized with unmodified OVA-LNP (**Figure 6A**). All animals showed no significant weight loss (**Figure S5**). When the tumor was allowed to grow until day 42, anti-IFNAR1 antibody treatment significantly abrogated the tumor growth control effect observed with the unmodified OVA-LNP in the isotype control group (**Figure 6B**). Mechanistically, anti-IFNAR1 antibody treatment reduced the expansion of splenic CD8^+^ T cell (**Figure 6C**) and antigen (OVA) specific IFN-γ-producing T cells (**Figure 6D**; **Figure S6**), compared with the isotype control treated group. Finally, we determined the phenotypes of tumor-infiltrating immune cells on day 42. Mice receiving anti-IFNAR1 antibody showed a significant increase in PD-1 expressing tumor-infiltrated CD4^+^ and CD8^+^ T cells (**Figure 6E**) and a significant increase in tumor-infiltrating M2-like macrophages (CD206^+^ F4/80^+^), compared to the isotype control treated group (**Figure 6F**). Overall, these results strongly indicated the crucial role of IFN-I signaling in unmodified mRNA-LNP-mediated anti-tumor immunity.

### Unmodified OVA-LNP suppresses metastasis to lung in a melanoma model

**Figure 7:**
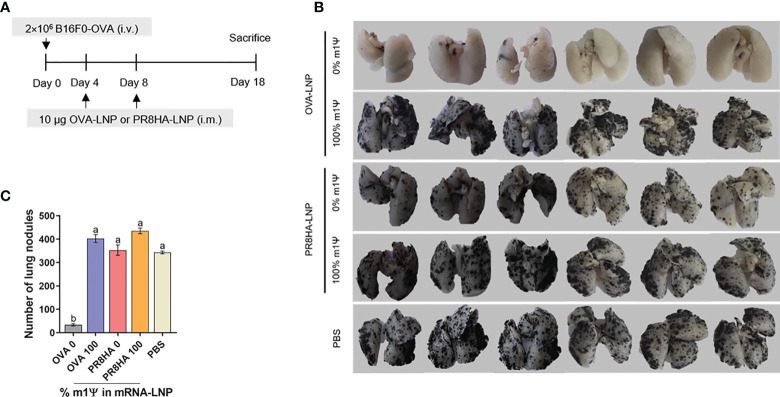
Immunization of unmodified mRNA-LNP inhibits lung metastasis of B16F0-OVA melanoma. **(A)** Schematic immunization schedule for melanoma metastatic model. See methods for details. **(B)** The lungs were observed and **(C)** the metastatic nodules on the surface of the lungs were counted. The results are presented as the mean ± SEM of biologically independent mice (*n* = 6) per group. Statistical significance by one-way ANOVA with Bonferroni multiple comparisons test when *p* < 0.05 compared to the unmodified target antigen: a, control: b.

Next, we tested whether unmodified OVA-LNP induces an immune response against lung metastasis in a melanoma model. B16F0-OVA cells were injected intravenously to establish lung metastasis. On day 4 and 8 after tumor cell injection, mice were i.m. immunized with two doses of OVA-LNP (10 μg/dose) with unmodified or m1Ψ modification of 100% or with unrelated antigen encoding mRNA-LNP (PR8HA-LNP) or PBS (**Figure 7A**). All animals reached endpoint without significant weight loss (**Figure S7**). On day 18, lung metastasis were observed and the number of lung nodules were counted (**Figures 7B, C**). The results showed that only unmodified OVA-LNP clearly suppressed nodule formation. In contrast, nucleoside modified OVA-LNP (100% m1Ψ modification) failed to control lung metastasis with comparable numbers of lung nodules as the PBS control or unrelated antigen (PR8HA-LNP). This result highlights the positive effect of antigen speicific unmodified mRNA-LNP on robust anti-tumor immunity including metastasis.

### Neoantigens (Pbk-Actn4) encoding mRNA-LNP is immunogenic

Based on the previous reports on mutanomes of B16F10 tumor (25, 26), we selected two of the somatic mutations of *Pbk* and *Actn4* as neoantigens to test in our study. These mutated epitopes of *Pbk* and *Actn4* were recognized and reacted to by CD8^+^ or CD4^+^ T cells, respectively, upon RNA monotope vaccinations and showed good MHC class I binding scores (‘low score’ 0.1 and 0.2, respectively) (25). These selected neoepitopes were linked with 10-mer non-immunogenic glycine/serine linkers and used as a neoantigen vaccine. To evaluate whether *Pbk*-*Actn4* encoding mRNA vaccines induce antigen-specific CD4^+^ and CD8^+^ T cell responses, mice were i.m. immunized with two doses of 10 µg/dose neoantigens (Neo-LNP) or control mCherry (mCherry-LNP) encoding mRNAs or PBS on day 0 and boosted with the same dose on day 4 (**Figure S8A**). Seven days after the boost, splenocytes were restimulated with overlapping peptide pools of *Pbk* and *Actn4*. Increased percentages of both CD4^+^ and CD8^+^ T cells producing IL-2, IFN-γ and TNF-α in the group receiving unmodified Neo-LNP were also observed (**Figures S8B, C**, **S9**). In addition, a significant increase in the frequencies of granzyme B and IFN-γ/granzyme B-producing CD8^+^ T cell were observed only in unmodified Neo-LNP (**Figures S8D**, **S10**). Taken together, epitope-specific CD4^+^ and CD8^+^ T cell responses were significantly induced upon immunization with unmodified Neo-LNP at a higher level, compared to that from Neo-LNP with m1Ψ modification of 100%.

### Neoepitope-specific immune responses induced by unmodified mRNA control B16F10 melanoma growth

**Figure 8:**
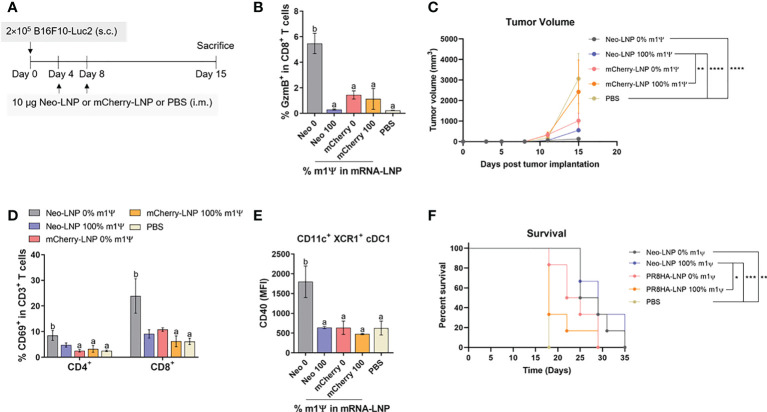
Neoantigens (Pbk-Actn4) encoding mRNA-LNP inhibits tumor growth *in vivo* and prolongs survival. **(A)** Schematic representation of the immunization regimen to test the anti-tumor efficacy of neoantigen encoding mRNA-LNP. See methods for details. **(B)** The frequency of GzmB-producing splenic CD8^+^ T cells on CD3^+^ CD8^+^ T cell subsets was determined by flow cytometry. **(C)** Tumor volume was measured (*n* = 10) **(D)** The frequencies of CD69^+^ T cells among tumor infiltrated CD4^+^ and CD8^+^ T cells are shown. **(E)** The MFI of costimulatory molecule CD40 on tumor-infiltrating cDC1 is shown (*n* = 4). **(F)** The percentage of survival was followed until day 35 (*n* = 6). GzmB, granzyme; cDC1, conventional type 1 dendritic cell. **(B)** The results are presented as the mean ± SEM. Statistical significance: (*)*p* < 0.05, (**)*p* < 0.01, (***)*p* < 0.001 and (****)*p* < 0.0001 by two-way ANOVA with Bonferroni multiple comparisons test. Survival curves were compared using log-rank (Mantel–Cox) test.

To examine the anti-tumor efficacy of Neo-LNP in B16F10 melanoma model, mice were i.m. immunized with two doses of Neo-LNP (10 μg/dose) or control mCherry-LNP (10 μg/dose) with unmodified and m1Ψ modification of 100% or PBS on day 4 and 8 after tumor implantation (**Figure 8A**). Repeated vaccination with the unmodified Neo-LNP in B16F10 tumour-bearing mice increased splenic antigen-specific granzyme B^+^ CD8^+^ T cells (**Figure 8B**; **Figure S8**). Consistent with the robust anti-neoantigen response, tumour growth was profoundly delayed and size/burden significantly decreased in unmodified Neo-LNP vaccinated group (**Figure 8C**). Mice given unmodified Neo-LNP showed a significant increase in CD69^+^ tumor-infiltrating T cells (**Figure 8D**; **Figure S9**) and the expression levels of CD40 in cDC1 (CD11c^+^ XCR1^+^) (**Figure 8E**; **Figure S9**). One third of the Neo-LNP treated mice survived until day 35, while all mice in the control group died by day 29 (**Figure 8F**). All animals reached endpoint termination without significant weight loss (**Figure S10**). Taken together, we confirmed that unmodified mRNA encoding tumor neoantigen formulated in LNP induced a strong anti-tumor immune response that retarded tumor growth and partially prolonged survival of tumor-bearing mice.

## Discussion

Recognition of uridine bases by innate immune sensors subsequently triggers cascades of innate immune responses that dictate the adaptive immune phenotypes. Substitution of uridine with m1Ψ in mRNA significantly improves the translation efficiency and decreases innate immunogenicity (20). Antitumor innate immune signals, particularly type I IFNs, which are the main cytokines secreted from DCs upon mRNA transfection (35) play an important role in antigen presentation and T cell differentiation into cytolytic effector cells. Herein, we addressed the impact of different m1Ψ percentages incorporated in mRNA on the immunogenicity and anti-tumor effects of the mRNA-LNP platform in B16 melanoma models using model antigen OVA and neoantigens.

cDC1 are critical for antigen cross-presentation required to prime CD8^+^ T cells for optimal anti-tumor immunity and priming of CD4^+^ T cells at early stages, partly because cDC1 provides antigen transportation to lymph nodes for processing by cDC2 (36). CD40 signaling in cDC1 is required for tumor rejection by playing a key role in augmenting the proliferation of antigen-specific CD8^+^ T cells (36). Engagement of CD40 with its ligand induces recruitment of TNF receptor-associated factor family of proteins (TRAFs) and initiates signaling cascades that activate genes involved in cytokine production, as well as upregulation of co-stimulatory molecules such as CD80 and CD86 (37). We demonstrated that the maturation of cDC1 and cDC2 upon delivery of unmodified and 40% modification with m1Ψ mRNA/LNP was evident compared with mRNA with 100% modification with m1Ψ. Intratumoral cDC1 also increased CD40 expression with the unmodified mRNA-LNP (**Figure 4E**). While unmodified mRNA/LNP compromises the translation efficiency of mRNA into protein antigen, its superior impact on DC maturation is beneficial for anti-tumor immunity.

Recent study identified the intrinsic adjuvant activity of the LNP itself. When used in mRNA and protein subunit vaccines, LNP exerts potent stimulatory activity against T follicular helper cell and the immune induction was superior to what induced by AddaVax formulated vaccine. Adjuvant activity of the LNP critically relies on IL-6 and its constituent ionizable lipid. Remarkably, potent immune responses from a single immunization of LNP loaded non-inflammatory nucleoside-modified mRNA was related to LNP adjuvanticity (38). Unmodified mRNA itself provides adjuvant activity through binding and activation of the innate immune sensors, mainly TLRs 3, 7, and 8 (39). In our study, we did not distinguish adjuvant activity of mRNA from LNP and the impact on anti-tumor responses may derive from LNP and/or mRNA.

Unmodified mRNA-LNP administration is associated with large amounts of systemic IFN-I at 6 h after immunization. Surprisingly, the level of IFN-I dramatically drops after the second dose of immunization which is likely the effect of unmodified mRNA. It is possible that repeated exposure to unmodified mRNA epigenetically enforces innate immune tolerance where the cells are incapable of activating certain inflammatory gene transcription (40).

The prominent therapeutic efficacy of unmodified mRNA is possibly due to activation of endosomal toll-like receptor 7/8 (TLR7/8) and subsequently causes pro-inflammatory cytokine secretion *via* MyD88-dependent IRF-5 phosphorylation (41). IRF-5 is critically involved in M1- macrophage polarization (42), which possesses phagocytic capacity, and the ability to secrete reactive nitrogen and oxygen species and pro-inflammatory cytokines such as IL-6, IL-12, IL-23 and TNF-α, which in turn promote CD8^+^ T cell and NK cell cytotoxicity. In addition, M1 macrophages secrete CXCL9, CXCL10 and CXCL15 chemokines upon STAT1 signaling, which recruit cytotoxic T lymphocytes (CTLs) to the tumor (43). Furthermore, the decrease of M2-like macrophages favors lung metastasis inhibition due to a lack of tumor-angiogenesis factors such as vascular endothelial growth factor (VEGF), epidermal growth factor (EGF) and fibroblast growth factor (FGF), and matrix metalloproteases (MMP-2 and MMP-9) which promote tumor angiogenesis, and metastasis (44).

Better tumor control with unmodified mRNA/LNP is associated with the presence of mature tumor-infiltrating migratory cDC1. The presence of mature cDC1 in tumor may lead to more efficient antigen presentation and cross-presentation of tumor antigens and subsequent augment antigen-specific T cell immunity (45) as shown in relevant results of antigen-specific effector CD8+ T cell (CD44^+^CD62L^-^) expansion and polyfunctional cytokine secretion after restimulated splenocytes with the OVA_257-264_ (SIINFEKL) peptide. Interestingly, we found that a substantial tumor infiltrated T cell subset (CD3^+^) in unmodified mRNA-LNP group is CD4^-^CD8^-^ double negative (DN) T cells (**Figure 4D**). Both TCRαβ T cells and TCRγδ T cells contain a small subset of DN T cells, suggesting both innate and adaptive functions. Althought the roles of these cells in tumors are still controversial, the use of DN T cells for cancer immunotherpy against blood and solid tumor were reported (46). Our results indicated that DN T cells may play a crucial role in anti-tumor immunity raised by mRNA vaccines. Therefore, characterization of the DN T cells may provide insight into the anti-tumor immunity induced by mRNA-LNP.

In the previous study by Kranz et al., a systemic immunization with three doses (40 µg/dose) of mRNA encoding OVA cleared B16-OVA lung metastasis with no tumor at 20 days after the last immunization. Their OVA mRNA construct encoded for the H-2K^b^-restricted immunodominant epitope OVA _257-264_ and the lipid formulation contained DOTAP and DOPE with the mean diameter of mRNA-LNP was 200 nm (30). In our study, using two doses (10 µg/dose) of mRNA encoding whole OVA protein, we observed a similar anti-metastatic effect. Although unmodified mRNA was used in the current work and that by Kranz et al., differences in the use of whole protein rather than peptide antigens, the LNP formulation and its size may result in the modest differences observed between the two reports. LNP used in our study is the proprietary to Acuitas Therapeutics, contains a proprietary ionizable cationic lipid, cholesterol, DSPC, and a PEG-lipid with a mean diameter of 80 nm (38).

In the tumor microenvironment, immunosuppression and tumor evasion strategies cause an inability of the immune cells to detect and eliminate with subsequent exhaustion (47). Generally, PD-1 expression on cell surface of activated T cells is induced after T cell receptor (TCR) activation (48). Ligation of PD-1 with its ligands programmed death-ligand 1 or 2 (PD-L1 or PD-L2) induces tyrosine phosphorylation of the PD-1 cytoplasmic domain by phosphorylating kinase Lck and subsequent recruitment of cytosolic tyrosine phosphatase SHP-2 and PD-1-associated SHP-2 preferentially dephosphorylates CD28 and suppresses CD28 costimulatory signaling leading to restrained effector T cell function (49–51). In our studies, we consistenly observed higher frequency of PD-1^+^ cells in tumor infiltrating CD4^+^ and CD8^+^ T cells when IFNAR1 was blocked. This result may imply that unmodified mRNA may help alleviate T cell exhaustion *via* IFN-I that allows anti-tumor T cells to be fully functional. Consistent with this observation, more M2-like tumor-associated macrophages were observed when IFN-I is blocked in the mRNA-LNP vaccinated group.

Accumulating data suggest that IFN-I strengthens antitumor T cell immunity by acting either indirectly or directly on T cells. IFN-I indirectly influences on T cell priming by upregulation of co-stimulatory molecules on APCs and directly acts as activating stimuli to prevent the abortive T-cell responses. Furthermore, IFN-I also exhibits direct stimulatory effect on immune cells by promoting IFN-γ secretion. Previous study showed that DC-specific *Ifnar* ^-/-^ mice were unble to reject highly immunogenic tumor cells due to the defects in antigen cross-presentation to CD8^+^ T cells. This evidence strongly shows that IFN-I can act through DCs to promote T cell immunity (52). Taken together, we provide strong evidence that IFN-I, directly or indirectly, through plays an indispensable role in inducing anti-tumor response by mRNA vaccine.

Although CD8^+^ T cells are known to play a pivotal role in antitumor immunity, CD4^+^ T cells also contribute to direct tumor killing besides their supporting role as cytokine producers. Previous study reported the observation of a cytotoxic subset of CD4^+^ T cells (CD4 CTLs). CD4^+^ CTLs are characterized by their cytotoxic functions to secrete granzyme B and perforin, two major tools to directly kill the target cells. CD4 CTL recognizes target cells *via* peptide-MHC II complex on APCs (53). Upon transferring of naive tumor reactive CD4^+^ T cells into lymphopenic recipients, substantial T cell expansion and differentiation were observed. Tumor regression was dependent on class II-restricted recognition of tumors by tumor-reactive CD4^+^ CTLs which developed cytotoxic activity and kill tumor (54).

For mRNA vaccine, modifying the structural elements of mRNA including the 5′ cap, 5′-and 3′-untranslated regions, the coding region, and polyadenylation tail help improved the intracellular stability of mRNA (55). Replacing of uridine by pseudouridine into mRNA gives superior nonimmunogenic mRNA with increased translational capacity and biological stability (20). Furthermore, lyophilization of modified mRNA-LNP provides long-term stability at room temperature (56). Whether unmodified mRNA-LNP shows similarly enhanced stability upon lyophilization is not known.

Finally, more relevant to real cancer settings with non-dominant antigens and tumor heterogeneity, Pbk-Actn4 somatic mutations of B16F10 tumor were selected and linked together as target neoantigens in mRNA-LNP vaccines. We observed less robust, but significant tumor growth retardation effect with the neoantigen vaccine compared to the OVA model. Additional neoatigens formulated in the mRNA vaccine may help improve the anti-tumor response of mRNA vaccines using neoantigens, such as demonstrated by Kreiter et al. (25). Taken together, we provide strong evidence for the anti-tumor immune response by unmodified mRNA vaccines encoding dominant and neoantigens.

## Data availability statement

The original contributions presented in the study are included in the article/**Supplementary Material**. Further inquiries can be directed to the corresponding author.

## Ethics statement

The animal study was reviewed and approved by The institutional animal care and use committee (IACUC) at the University of Pennsylvania and Chulalongkorn University.

## Author contributions

CS, M-GA, DW and TP: study conception and design, CS, M-GA: data collection, CS, M-GA and TP: analysis and interpretation of results, PL and YT: reagent preparation and analysis, CS and TP: draft manuscript preparation. EP and TP: grant funding acquisition. All authors contributed to the article and approved the submitted version.

## Funding

This study was supported in part by the Chulaongkorn Academic Advancement into its Second Century (CUAASC) Project, Ratchadaphiseksomphot Endowment Fund, by the National Research Council of Thailand and by the National Science, Research and Innovation Fund (NSRF) via Program Management Unit for Human Resources amp; Institutional Development, Research and Innovation (PMU-B) Grant No. B16F640117. CS received a scholarship from Science Achievement Scholarship of Thailand (SAST) and also The 90th Anniversary of Chulalongkorn University Fund (Ratchadaphiseksomphot Endowment Fund).

## Conflict of interest

PL and YT are employees of Acuitas Therapeutics, a company involved in the development of mRNA-LNP therapeutics. YT, DW, and M-GA are named on patents that describe lipid nanoparticles for delivery of nucleic acid therapeutics, including mRNA and the use of modified mRNA in lipid nanoparticles as a vaccine platform.

The remaining authors declare that the research was conducted in the absence of any commercial or financial relationships that could be construed as a potential conflict of interest.

## Publisher’s note

All claims expressed in this article are solely those of the authors and do not necessarily represent those of their affiliated organizations, or those of the publisher, the editors and the reviewers. Any product that may be evaluated in this article, or claim that may be made by its manufacturer, is not guaranteed or endorsed by the publisher.

## Supplementary material

Supplementary Figure 1 Flow gating strategies. **(A)** Representative flow gating strategy of translation efficiency of mRNA-LNP and BMDC maturation for and 1f is shown. **(B)** Representative flow gating strategy of translation efficiency of mRNA-LNP in BMDM for is shown. **(C)** Representative flow gating strategy of translation efficiency of mRNA-LNP and APC maturation in lymphoid organs for is shown. **(D)** Representative flow gating strategies of antigen-specific splenic CD8^+^ T cell response induced by mRNA-LNP for are shown.

Supplementary Figure 2 Effect of immunization of OVA-LNP on body weight changes in tumor bearing mice. Percent of body weight changes of B16F0-OVA tumor-bearing mice which received OVA mRNA-LNP is shown.

Supplementary Figure 3 Flow gating strategies. Representative flow gating strategies of tumor-infiltrating immune cell profiles for are shown.

Supplementary Figure 4 Flow gating strategies. Representative flow gating strategies of antigen-specific CD8+ T cells for are shown.

Supplementary Figure 5 Effect of immunization of OVA-LNP in tumor-bearing mice in the presence of IFN-I blocking on body weight changes. Percent of body weight changes of B16F0-OVA tumor-bearing mice that were immunized with OVA mRNA-LNP in the absence or presence of type I IFN neutralizing antibody is shown.

Supplementary Figure 6 ELISpot for IFN-γ in splenic T cells receiving mRNA-LNP in the presence of IFN-I blocking. Representative IFN-γ ELISpot of OVA-specific splenic T cell response is shown.

Supplementary Figure 7 Effect of immunization of OVA-LNP on weight loss in a melanoma metastasis model. Percent of body weight changes of mice implanted with B16F0-OVA tumor for lung metastasis and OVA mRNA-LNP is shown.

Supplementary Figure 8 Immunization of neoantigens (Pbk-Actn4) encoding mRNA-LNP elicits robust antigen-specific T cell responses. **(A)** Schematic representation of the immunization regimen. On days 0 and 4, mice were intramuscularly immunized with 10 µg of Neo-LNP constituting 0, or 100% of m1Ψ. Mice were treated with mCherry-LNP or PBS as controls. Mice were sacrificed on day 11 (7 days after the boost) and spleens were collected. Frequencies of cytokines-producing **(B)** CD4^+^ and **(C)** CD8^+^ T cells on CD3^+^ T cell subsets determined by flow cytometry after 6 h stimulation with pool of Pbk and Actn4 peptides at 7 days post-boost. **(D)** Frequencies of GzmB (left) and IFN-γ/GzmB (right)-producing CD8^+^ T cells on CD3^+^ CD8^+^ T cell subsets were determined by flow cytometry. GzmB, granzyme B. Each column are represented as the mean ± SEM, *n* = 4 biologically independent mice per group. Statistical significance by one-way ANOVA with Bonferroni multiple comparisons test when *p* < 0.05 compared to the unmodified target antigen: a, control (PBS): b.

Supplementary Figure 9 Flow gating strategies. Representative flow gating strategies of tumor-infiltrating immune cell profiles for are shown.

Supplementary Figure 10 Flow gating strategies. Representative flow gating strategies of tumor-infiltrating immune cell profiles for are shown.

Supplementary Figure 11 Effect of immunization of Neo-LNP on body weight changes in tumor bearing mice. Percent of body weight changes of B16F10-Luc2 tumor-bearing mice which was immunized with Neo mRNA-LNP is shown. Each dot are represented as the mean ± SEM, *n* = 4-9 biologically independent mice per group.

Supplementary Table 1 List of antibodies.
