# Supplementary material for: mRNA vaccine with unmodified uridine induces robust type I interferon-dependent anti-tumor immunity in a melanoma model
Source: Front Immunol. 2022 Oct 14;13:983000. doi: 10.3389/fimmu.2022.983000 (PMC9614103; doi:10.3389/fimmu.2022.983000)
Supplement: Supplementary Figure 1 — Flow gating strategies. (A) Representative flow gating strategy of translation efficiency of mRNA-LNP and BMDC maturation for and 1f is shown. (B) Representative flow gating strategy of translation efficiency of mRNA-LNP in BMDM for is shown. (C) Representative flow gating strategy of translation efficiency of mRNA-LNP and APC maturation in lymphoid organs for is shown. (D) Representative flow gating strategies of antigen-specific splenic CD8+ T cell response induced by mRNA-LNP for are shown. [file DataSheet_1.pdf]

Representative FACS panel for Figure 1a, 1e (left) and 1f

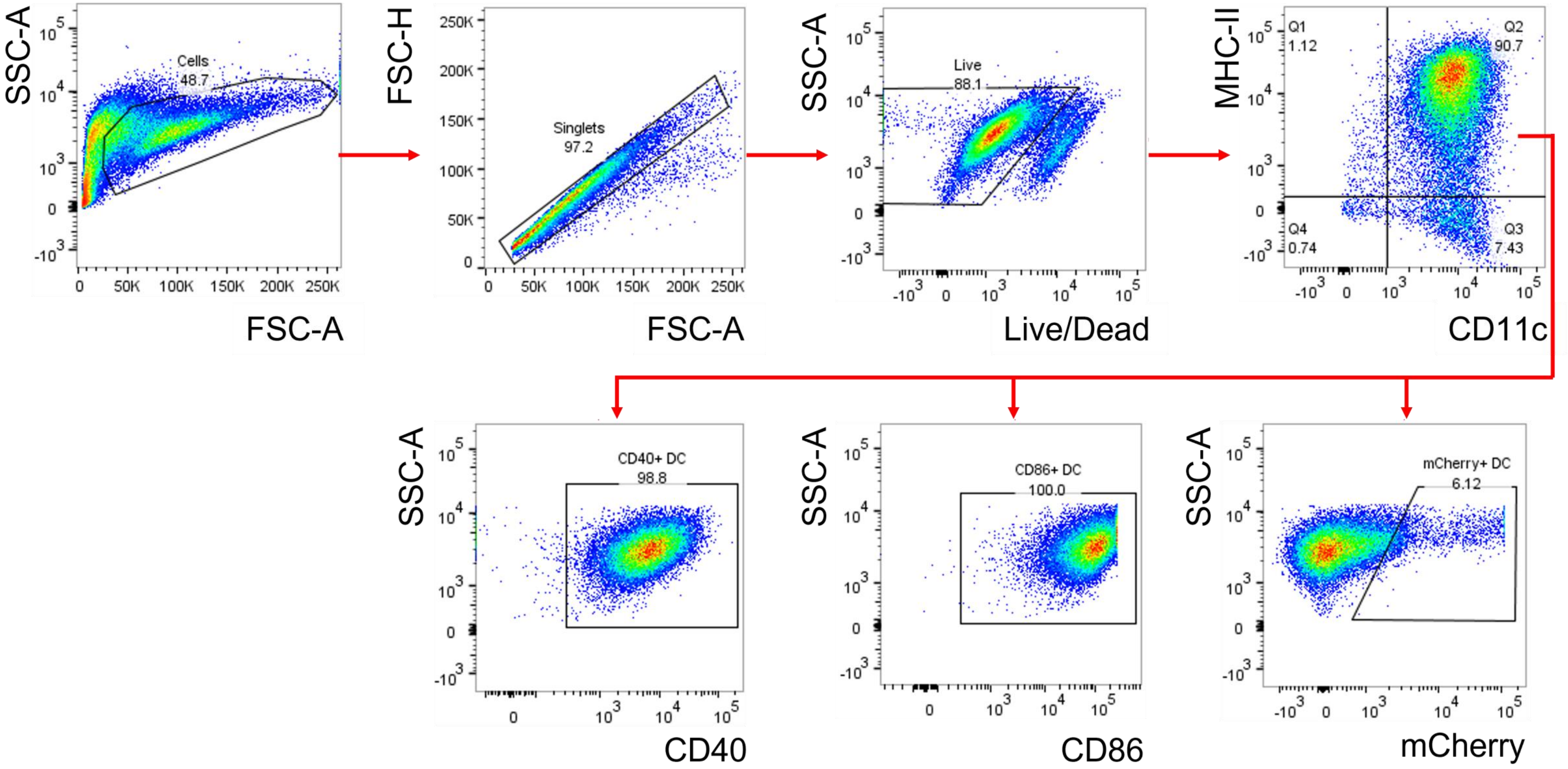

Supplementary Figure S1a
Sittplangkoon et al., 2022

Representative FACS panel for Figure 1b and 1e (right)

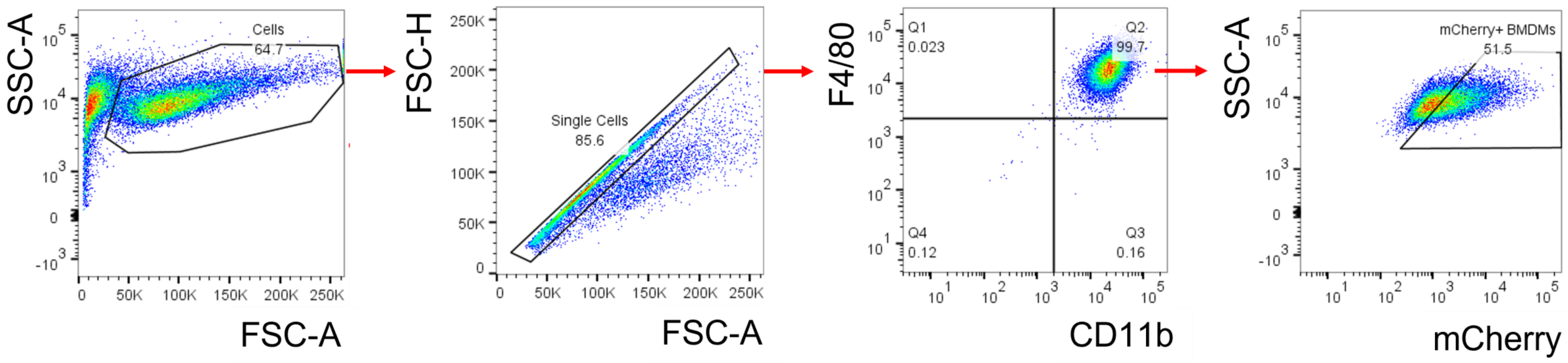

Supplementary Figure S1b
Sittplangkoon et al., 2022

Representative FACS panel for Figure 2

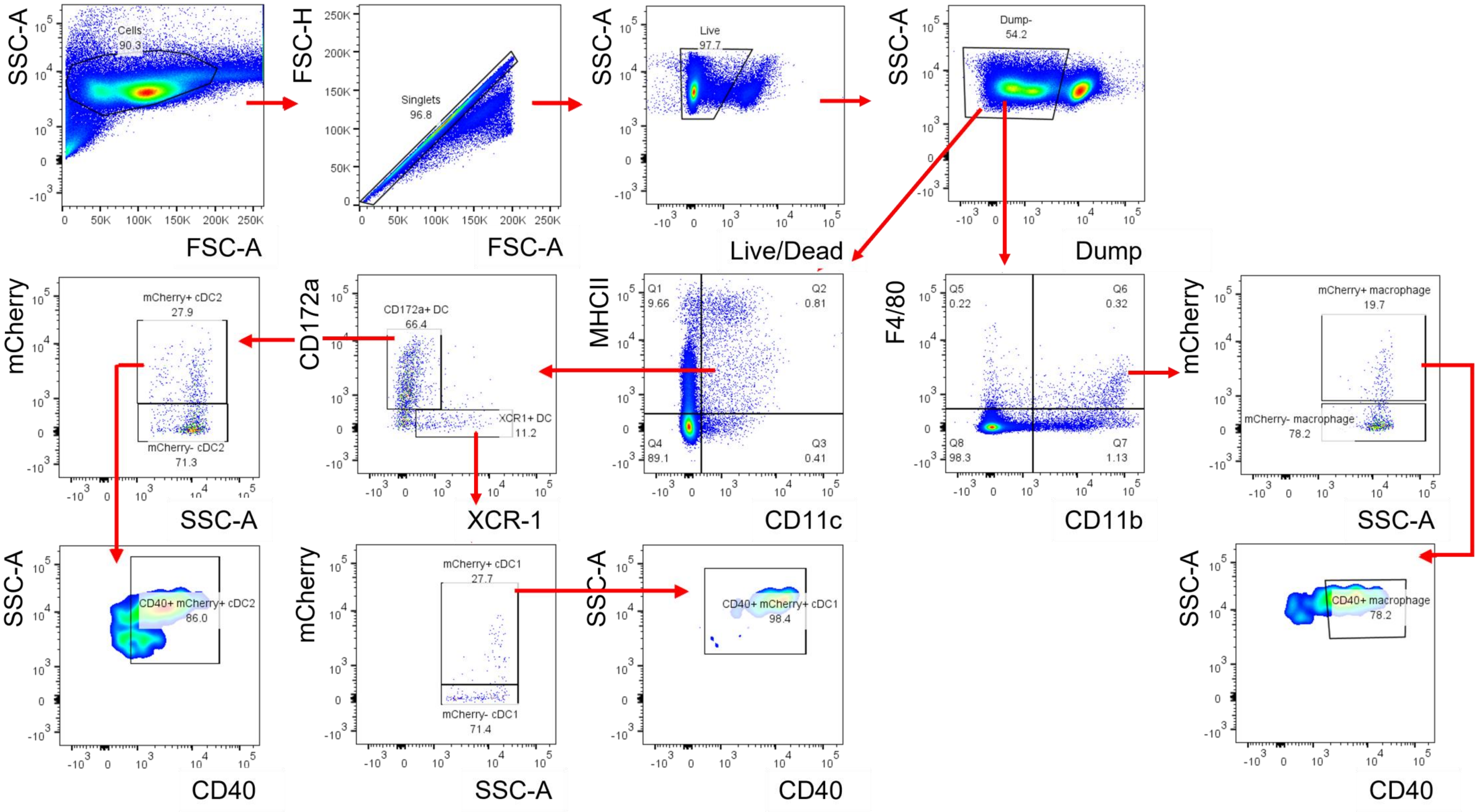

Supplementary Figure S1c
Sittplangkoon et al., 2022

Representative FACS panel for Figure 3

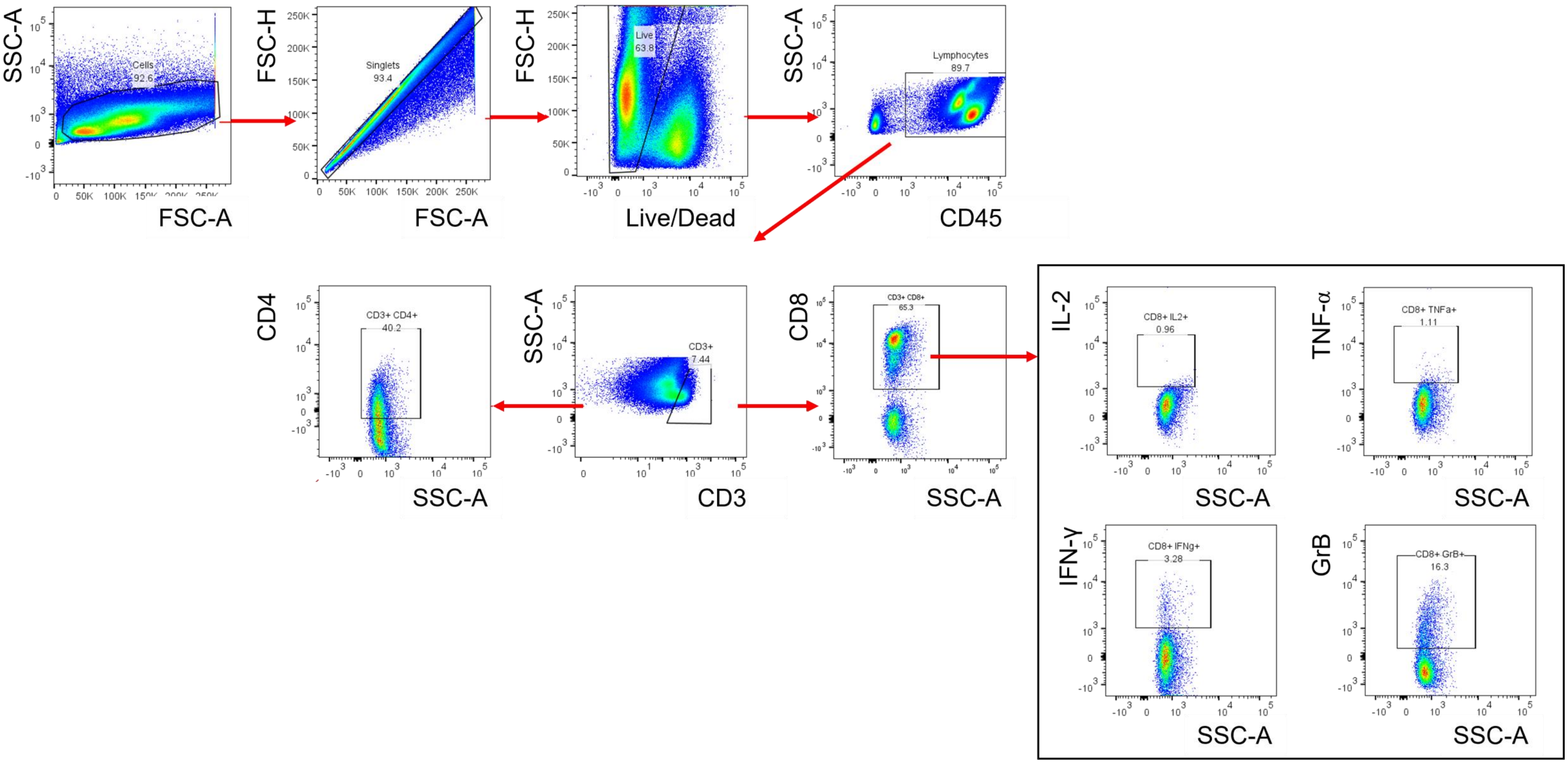

Supplementary Figure S1d
Sittplangkoon et al., 2022

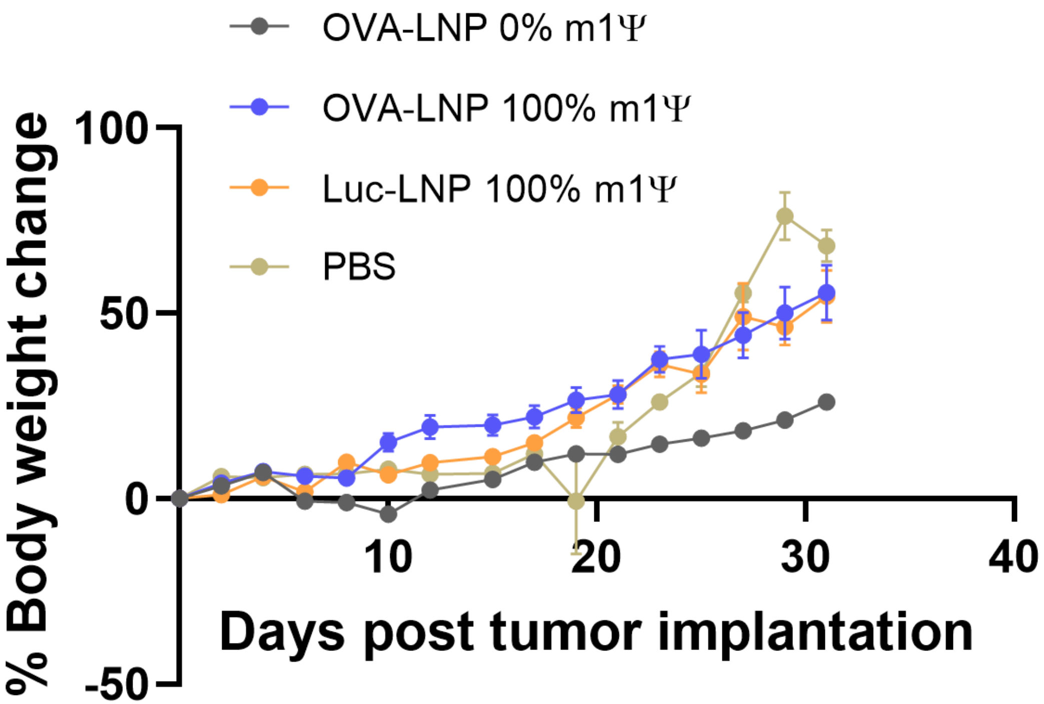

Supplementary Figure S2
Sittplangkoon et al., 2022

Representative FACS panel for Figure 4

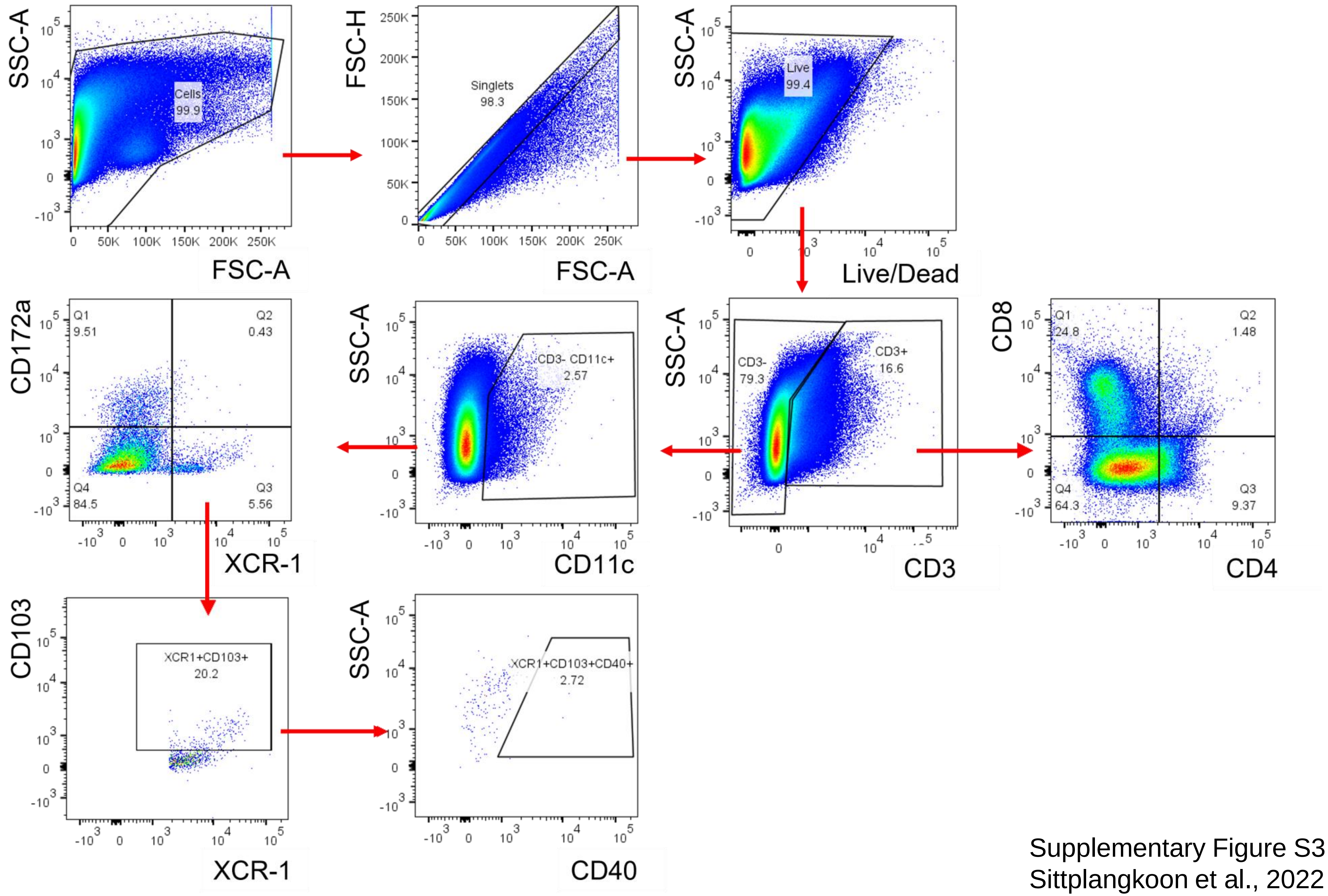

Supplementary Figure S3
Sittplangkoon et al., 2022

Representative FACS panel for Figure 5

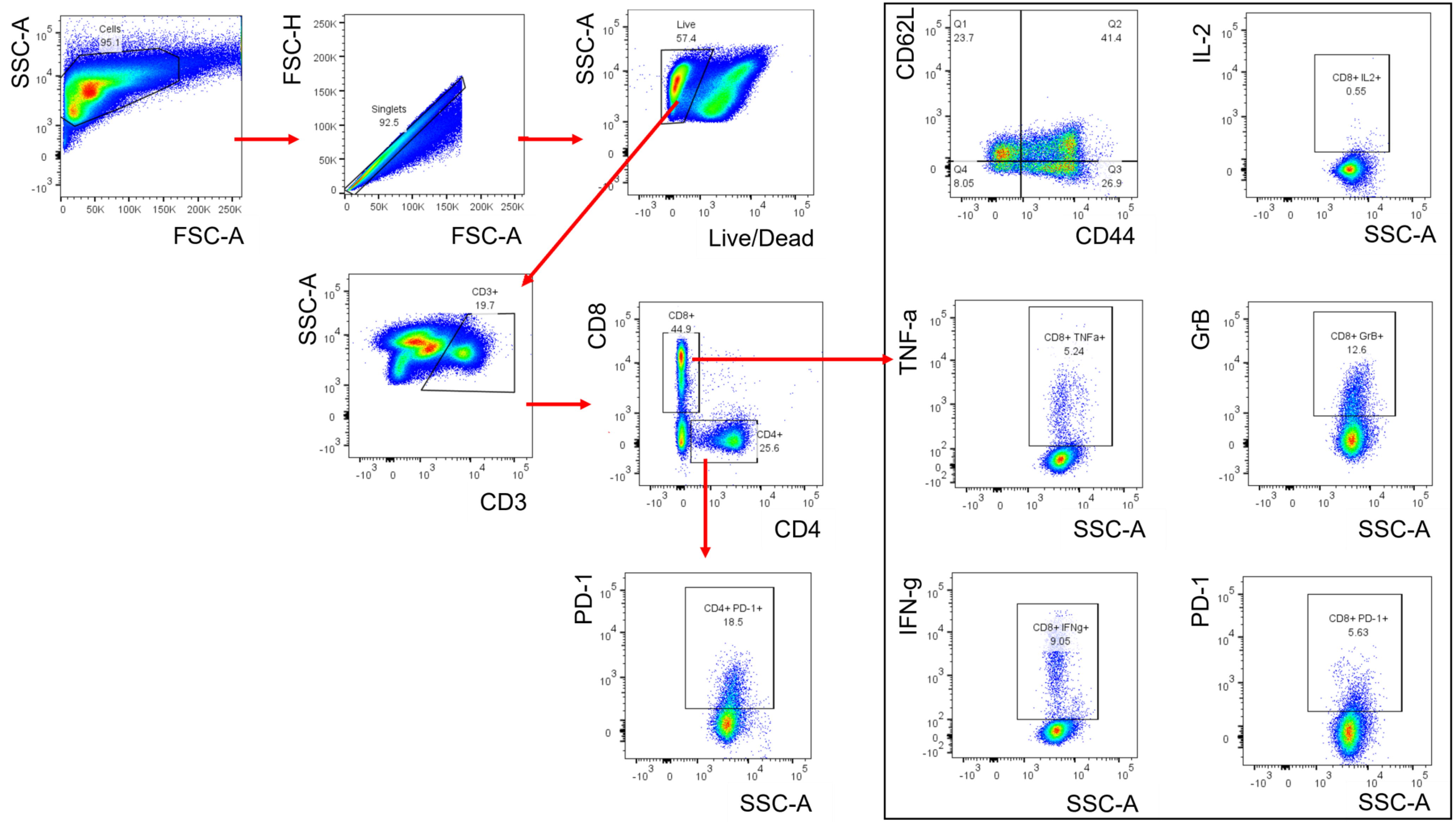

Supplementary Figure S4
Sittplangkoon et al., 2022

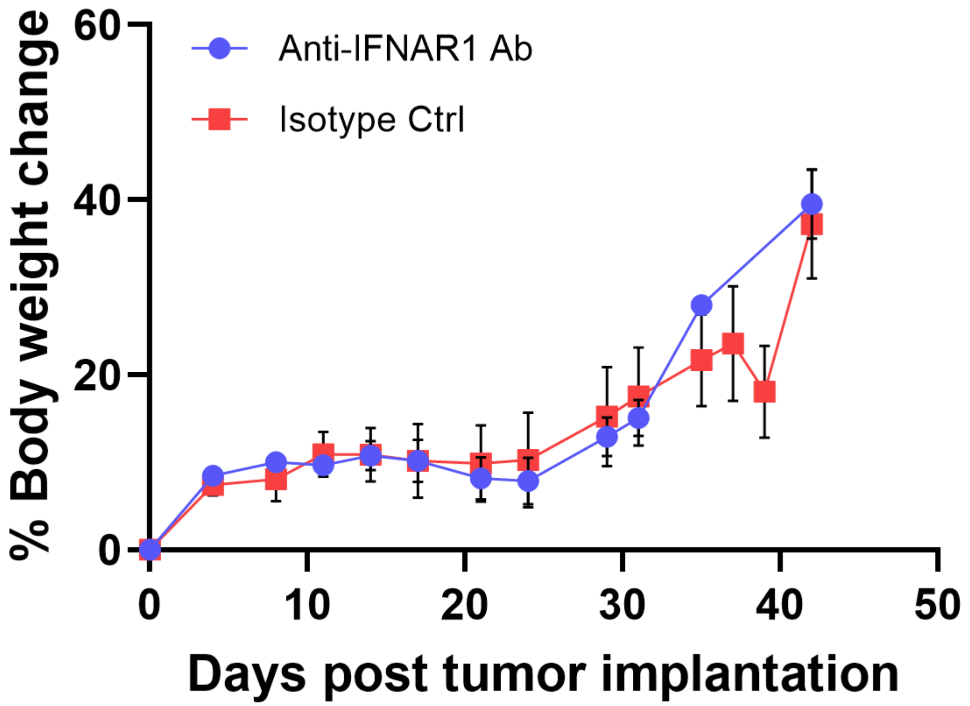

Supplementary Figure 5
Sittplangkoon et al., 2022

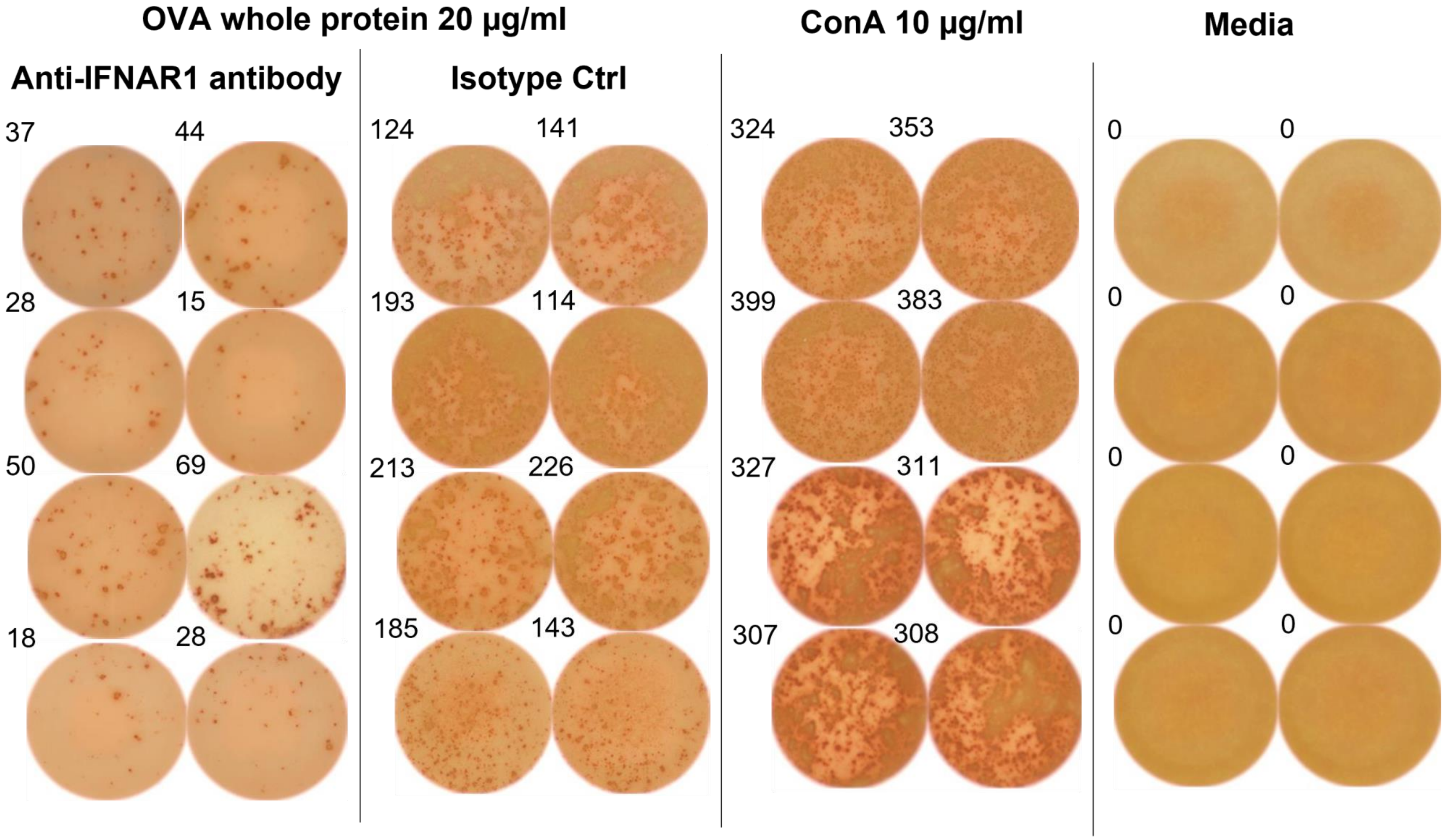

Supplementary Figure S6
Sittplangkoon et al., 2022

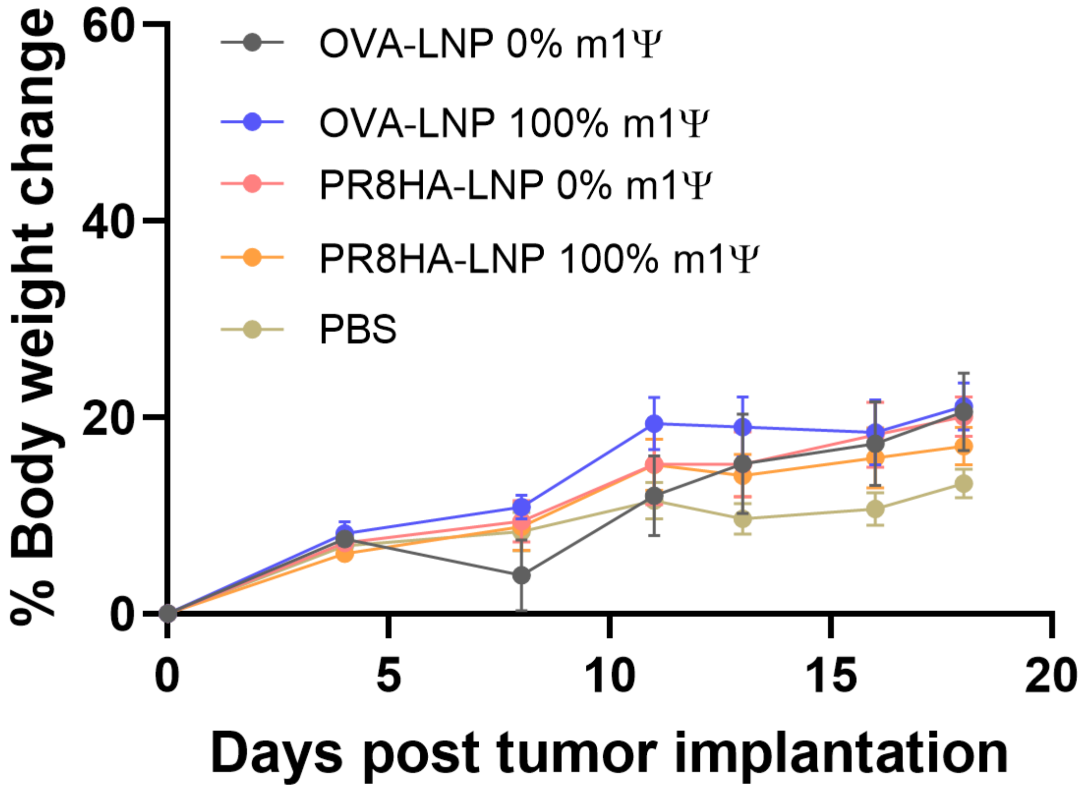

Supplementary Figure 7
Sittplangkoon et al., 2022

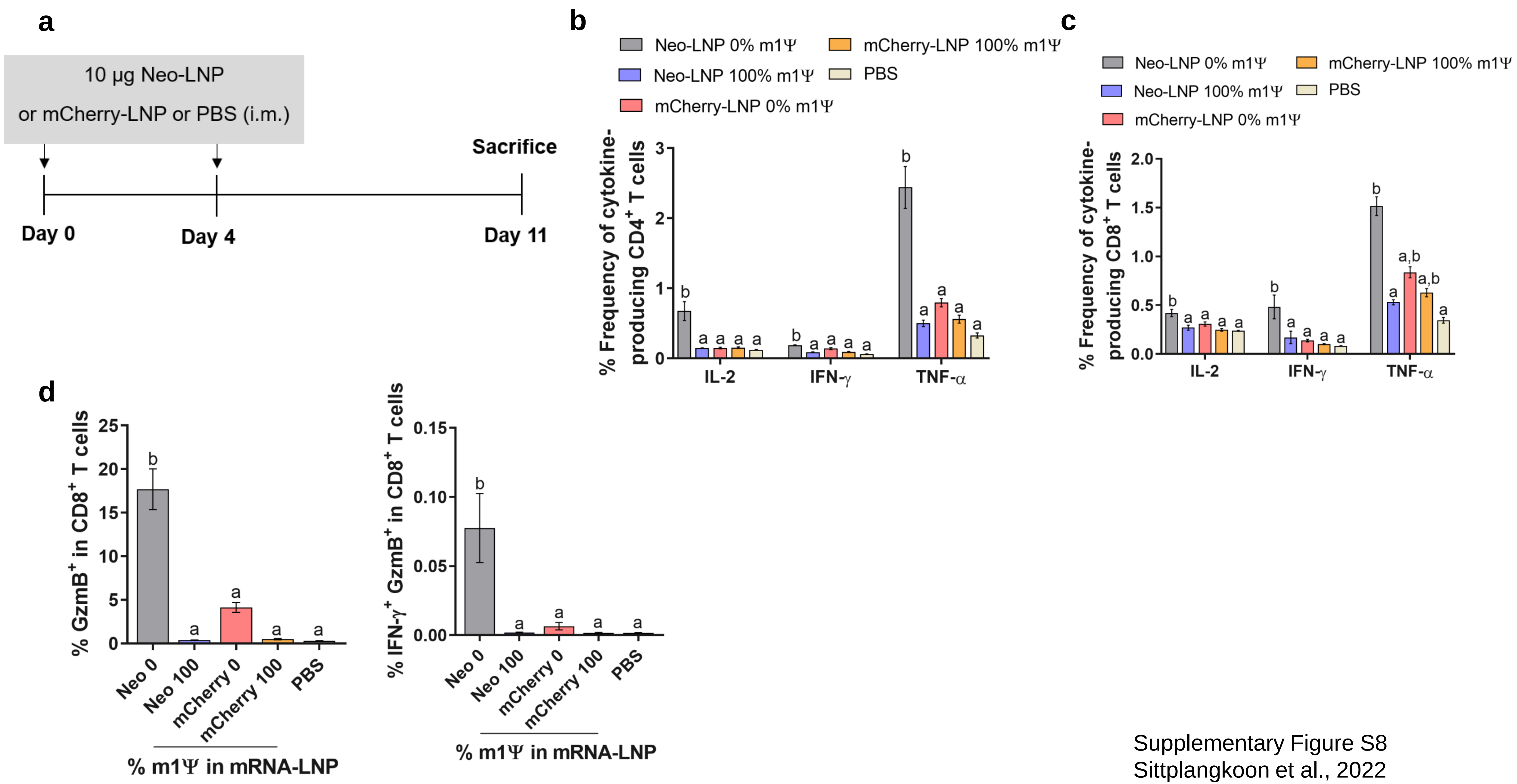

Supplementary Figure S8
Sittplangkoon et al., 2022

Representative FACS panel for Figure 8b

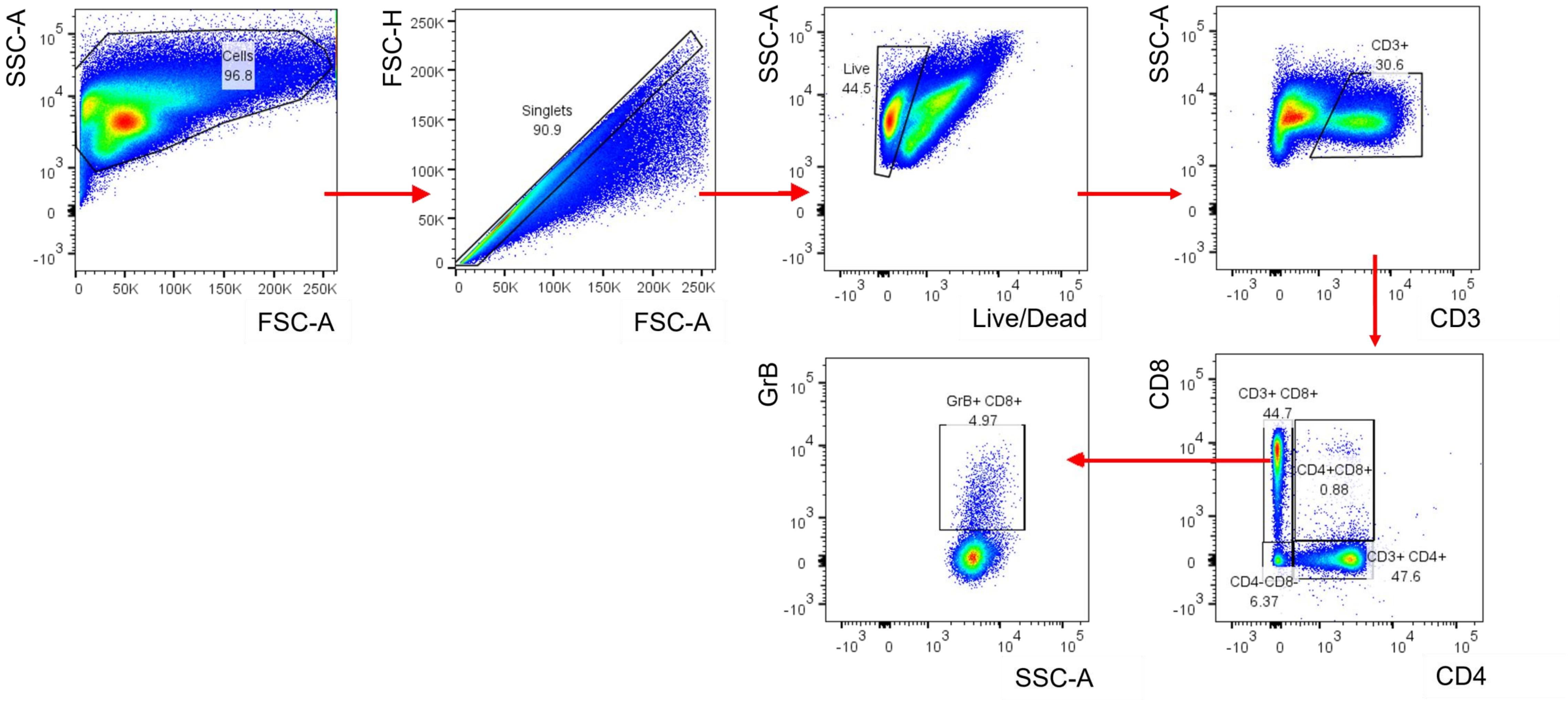

Supplementary Figure S9
Sittplangkoon et al., 2022

Representative FACS panel for Figure 8d and 8e

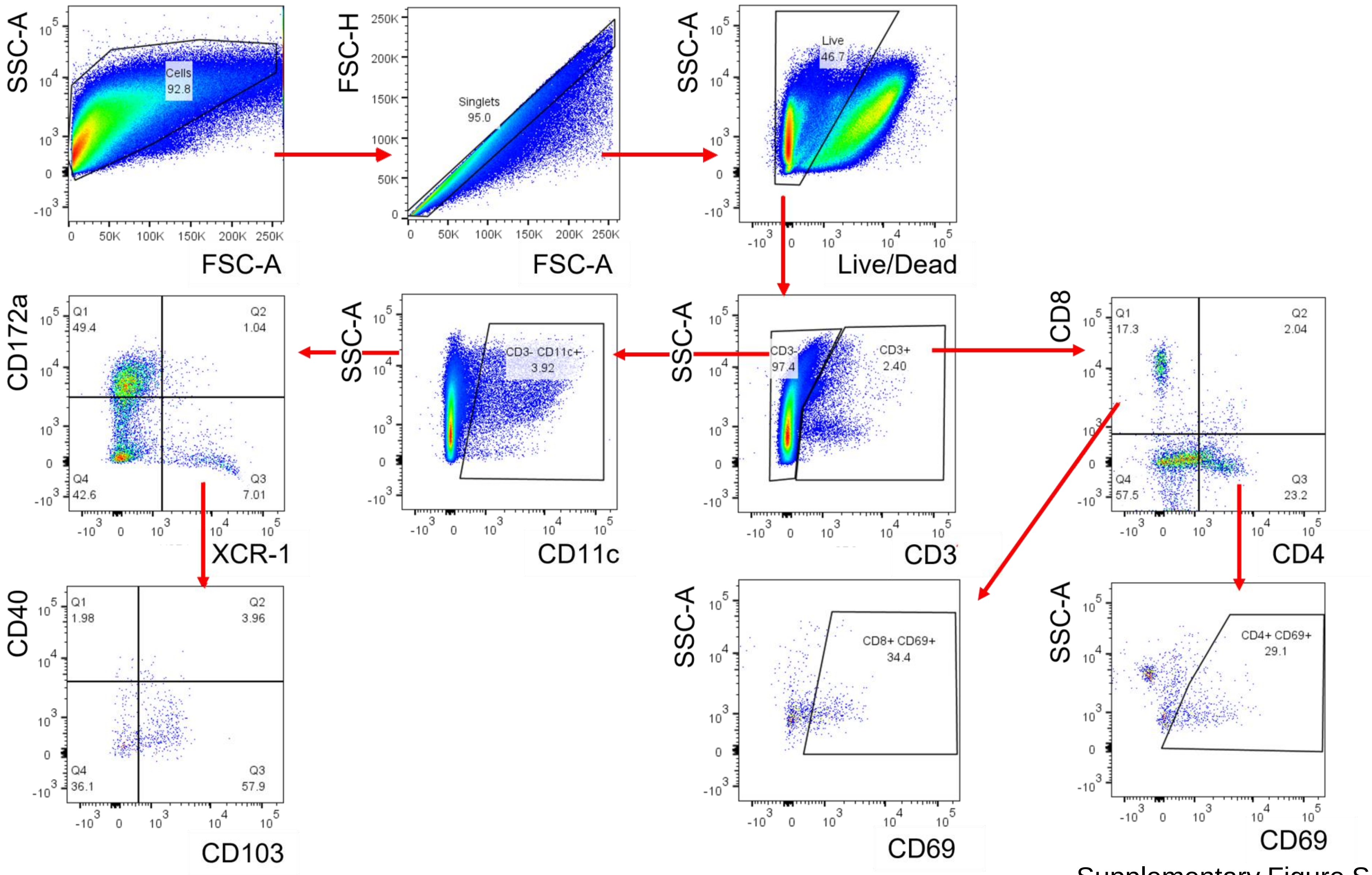

Supplementary Figure S10
Sittplangkoon et al., 2022

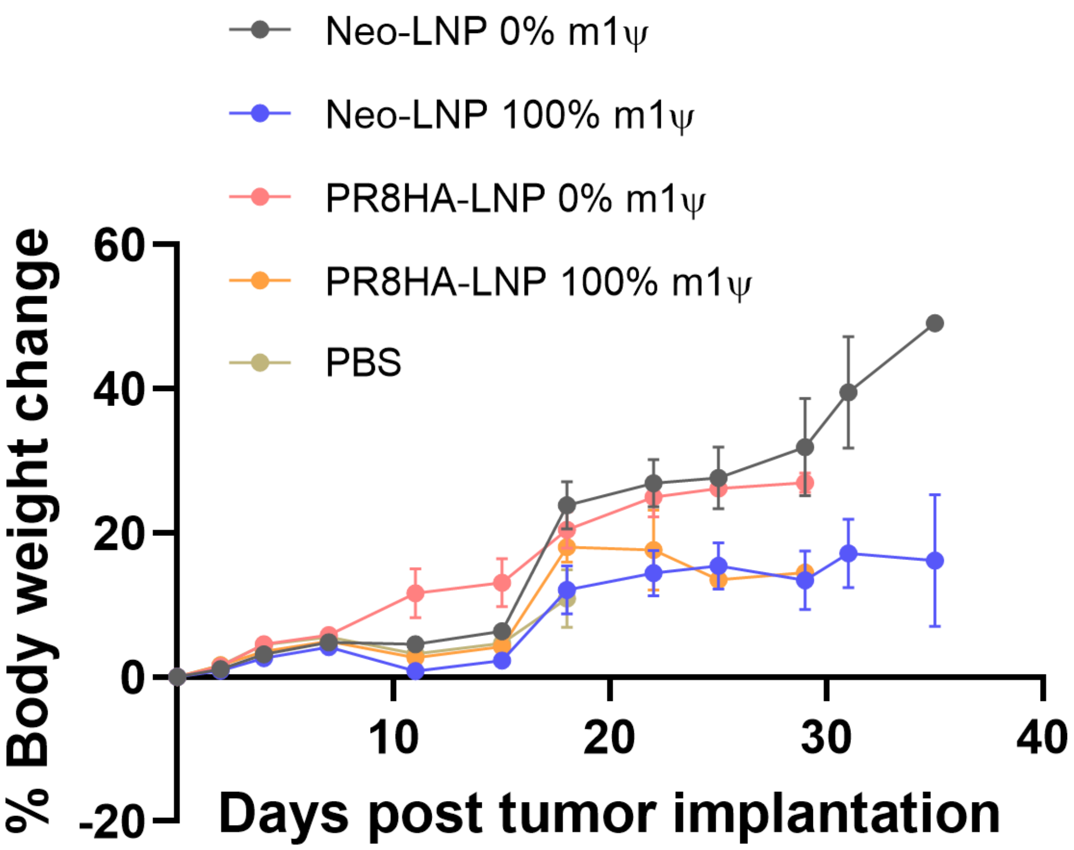

Supplementary Figure S11
Sittplangkoon et al., 2022
