# Supplementary material for: mRNA vaccine with unmodified uridine induces robust type I interferon-dependent anti-tumor immunity in a melanoma model
Source: Front Immunol. 2022 Oct 14;13:983000. doi: 10.3389/fimmu.2022.983000 (PMC9614103; doi:10.3389/fimmu.2022.983000)
Supplement: Supplementary Table 1 — List of antibodies. [file Table_1.docx]

**Supplementary Table 1**. List of antibodies

| **Antibody/clone/fluorophore** | **Source** | **Catalog number** | **Amount used per 2 million cells in 100 µl** |
| --- | --- | --- | --- |
| CD3, clone 17A2, biotin | BioLegend, USA | 100244 | 0.25 µg |
| CD3, clone 17A2, FITC | BioLegend, USA | 100204 | 0.5 µg |
| CD3, clone 17A2, BV605 | BioLegend, USA | 100237 | 0.25 µg |
| CD4, clone GK1.5, AF488 | BioLegend, USA | 100423 | 0.25 µg |
| CD4, clone GK1.5, PerCP/Cy5.5 | BioLegend, USA | 100434 | 0.2 µg |
| CD8, clone 53-6.7, PE | BioLegend, USA | 100708 | 0.25 µg |
| CD8, clone 53-6.7, BV785 | BioLegend, USA | 100750 | 0.2 µg |
| CD44, clone IM7, BV650 | BioLegend, USA | 103049 | 0.2 µg |
| CD62L, clone MEL-14, AF700 | BioLegend, USA | 104426 | 0.5 µg |
| PD-1, clone RMP1-30, PE/Cy7 | BioLegend, USA | 109110 | 0.25 µg |
| PD-1, clone 29F.1A12, APC/Cy7 | BioLegend, USA | 135224 | 0.2 µg |
| CD11b, clone M1/70, PE | BioLegend, USA | 101208 | 0.25 µg |
| F4/80, clone BM8, AF488 | BioLegend, USA | 123120 | 0.25 µg |
| CD206, clone C068C2, PE | BioLegend, USA | 141706 | 0.25 µg |
| IL-5, clone TRFK5, PE | BioLegend, USA | 504304 | 0.2 µg |
| Granzyme B, clone GB11, Pacific Blue | BioLegend, USA | 515408 | 2.5 µl |
| IFN-γ, clone XMG1.2, FITC | BioLegend, USA | 505806 | 0.5 µg |
| IFN-γ, clone XMG1.2, AF700 | BioLegend, USA | 505824 | 0.5 µg |
| IL-2, clone JES6-5H4, APC | BioLegend, USA | 503810 | 0.2 µg |
| TNF-α, clone MP6-XT22, PE/Cy7 | BD Pharmingen, USA | 557644 | 0.2 µg |
| CD69, clone H1.2F3, APC | BioLegend, USA | 104514 | 0.2 µg |
| CD40, clone 3/23, PE/Cy5 | BioLegend, USA | 124618 | 0.2 µg |
| CD86, clone GL-1, biotin | BioLegend, USA | 105004 | 0.25 µg |
| CD45R, clone I3/2.3, PC7 | Beckman Coulter, USA | A88587 | 0.25 µg |
| CD11c, clone N418, BV711 | BioLegend, USA | 117349 | 0.2 µg |
| XCR1, clone ZET, BV650 | BioLegend, USA | 148220 | 0.2 µg |
| CD103, clone 2E7, APC/Cy7 | BioLegend, USA | 121432 | 0.2 µg |
